# Supplementary material for: VISTA drives pancreatic tumor progression through modulation of the tumor-associated macrophage polarity
Source: Nat Commun. 2026 Mar 3;17:4582. doi: 10.1038/s41467-026-70215-7 (PMC13194722; doi:10.1038/s41467-026-70215-7)
Supplement: Supplementary file 1 — Supplementary Information [file 41467_2026_70215_MOESM1_ESM.pdf]

Supplementary Information

VISTA Drives Pancreatic Tumor Progression Through Modulation of the Tumor-Associated Macrophage Polarity

Suk-Kyung Shin,[1,2,3,4,#] Gwanghun Kim,[1,5,#] Su Min Park,[1,2,3,4] Eun-Bi Seo,[1,6] Sang-Kyu Ye,[1,2,4,6] Gyeong Hoon Kang,[7] Keehoon Jung,[1,4] Hyun Mu Shin,[1,2,4,5,*] Hang-Rae Kim,[8,9,*] Dong-Sup Lee, [1,3,4*]

[1]Department of Biomedical Sciences, Cancer Research Institute, Seoul National University College of Medicine, Seoul 03080, Republic of Korea

[2]Wide River Institute of Immunology, Seoul National University, Gangwon 25159, Republic of Korea

[3]Convergence Research Center for Dementia, Seoul National University Medical Research Center, Seoul 03080, Republic of Korea

[4]BK21 FOUR Biomedical Science Project, Seoul National University College of Medicine, Seoul 03080, Republic of Korea

[5]Medical Research Center, Seoul National University College of Medicine, Seoul 03080, Republic of Korea

[6]Department of Pharmacology, Ischemic/Hypoxic Disease Institute, Seoul National University College of Medicine, Seoul 03080, Republic of Korea

[7]Department of Pathology, Seoul National University College of Medicine, Seoul 03080, Republic of Korea

[8]Samsung Precision Genome Medicine Institute, Research Institute for Future Medicine, Samsung Medical Center, Seoul 06351, Republic of Korea

[9]Department of Health Sciences and Technology, Samsung Advanced Institute for Health Sciences & Technology (SAIHST), Sungkyunkwan University, Seoul 06355, Republic of Korea

[#]Equally Contributing Authors

[*]Corresponding authors

This document includes:

- Supplementary Figures 1 to 22
- Supplementary Tables 1 to 4

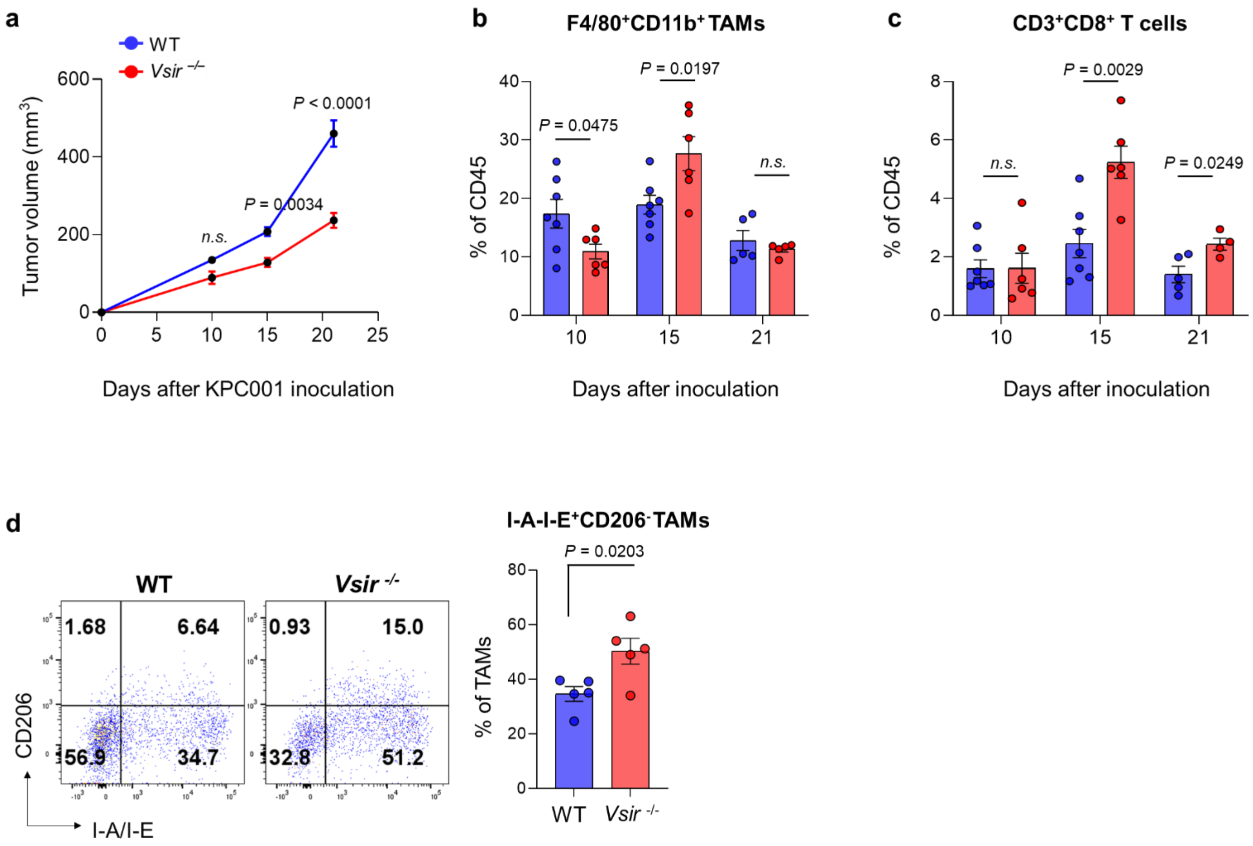

Supplementary Fig. 1 | VISTA deficiency reduces tumor growth in KPC001 model. a Tumor growth curves of wild-type (WT) and *Vsir*$^{-/-}$ mice orthotopically implanted with KPC001 cells (WT, $n = 42$, *Vsir*$^{-/-}$, $n = 42$). Statistical significance at each time point was determined using two-way ANOVA followed by Sidak's multiple-comparisons test (two-sided). **b** Flow cytometric quantification of F4/80^{+}CD11b^{+} tumor-associated macrophages (TAM) in WT ($n = 19$) and *Vsir*$^{-/-}$ ($n = 17$) mice at days 10, 15, and 21, shown as a percentage of CD45^{+} cells. **c** Flow cytometric quantification of CD8^{+} T cells in WT ($n = 19$) and *Vsir*$^{-/-}$ ($n = 16$) mice at days 10, 15, and 21, shown as a percentage of CD45^{+} cells. **d** Flow cytometric analysis of I-A/I-E^{+} CD206^{-} TAMs in tumor, expressed as a percentage of CD45^{+} cells ($n = 5$). All data are presented as mean ± SEM. For panels **b**–**d**, statistical significance was determined using unpaired two-sided Student's *t*-tests. Exact *P* values are shown in the figures. *n.s.*, not significant. Experiments were independently repeated at least three times with similar results.

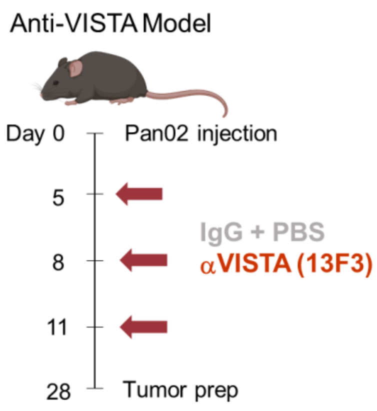

Supplementary Fig. 2 | Anti-VISTA (αVISTA) antibody treatment schedule. Pan02 cells were orthotopically inoculated into wild-type (WT) mice. Anti-VISTA antibody was administered intraperitoneally on days 5, 8, and 11 after tumor inoculation.

Created in BioRender. Kim, H. (2026) https://BioRender.com/r91s305

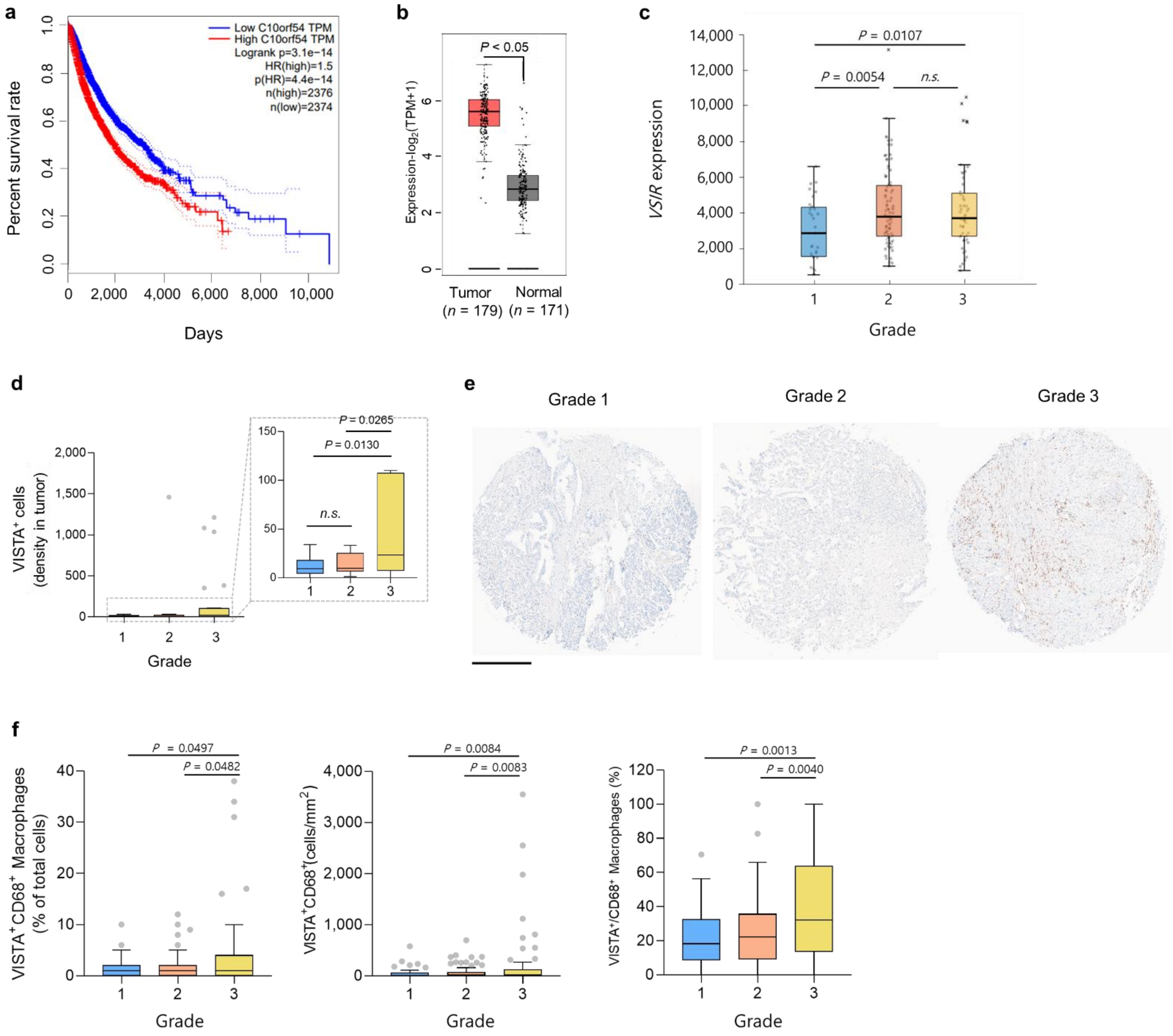

Supplementary Fig. 3 | High *VSIR* (c10orf54) expression level correlates with poor overall survival in PAAD patients. a Kaplan-Meier survival analysis of the *VSIR* signature in The Cancer Genome Atlas (TCGA) pancreatic adenocarcinoma (PAAD) cohort. Statistical significance was determined using a two-sided log-rank (Mantel-Cox) test. Dashed lines indicate 95% confidence intervals. Vertical tick marks represent right-censored data points. **b** *VSIR* expression levels in tumor (n = 179) *versus* normal pancreas (n = 171) samples from the TCGA PAAD cohort, transformed as –log(TPM + 1). Data are presented as box-and-whisker plot, along with gray circles representing outliers defined by Tukey's fence method. Statistical significance was determined using a two-sided Wilcoxon rank-sum test. P value was adjusted for multiple comparisons using the Benjamini–Hochberg method (FDR-adjusted P value). **c** *VSIR* expression levels stratified by tumor grade

in the TCGA PAAD cohort. Statistical significance was assessed using ANCOVA analysis ($P = 1.36 \times 10^{-8}$). **d** Quantification of VISTA$^+$ cells in pancreatic tumors stratified by tumor grade (Grade 1, $n = 7$; Grade 2, $n = 4$; Grade 3, $n = 18$). Data are presented as box-and-whisker plot, along with gray circles representing outliers defined by Tukey's fence method. Statistical significance was determined using the Kruskal-Wallis test followed by Tukey-Kramer multiple-comparison test (two-sided). **e** Representative immunohistochemical images from a tumor microarray. Scale bar, 600 μm. **f** Percentage and cell density of VISTA$^+$CD68$^+$ co-expressing macrophages (left and middle), and the proportion of VISTA$^+$ cells within the CD68$^+$ macrophage population (right), across tumor grades in tissue microarrays. Data are presented as box-and-whisker plot, along with gray circles representing outliers defined by Tukey's fence method. Statistical significance was determined using one-way ANOVA followed by Tukey's multiple-comparisons test (two-sided). Exact *P* values are shown in the figure. *n.s.,* not significant.

a Gating strategy referred to Figure 1d

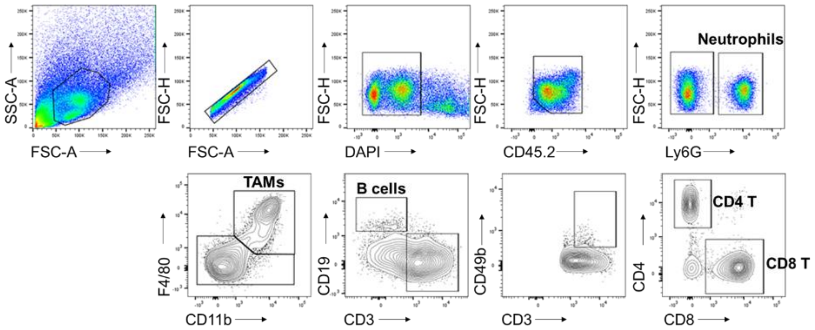

b Gating strategy referred to Figure 1f and 1g

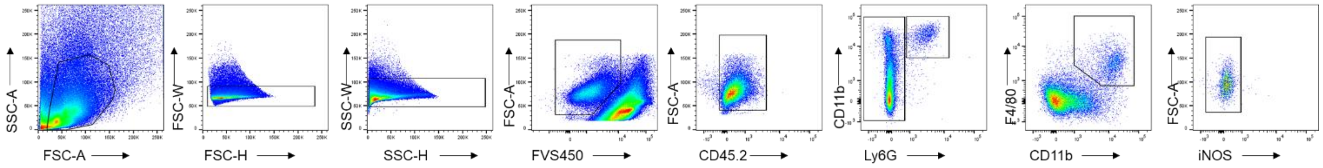

c Gating strategy referred to Supplementary Figure 6b

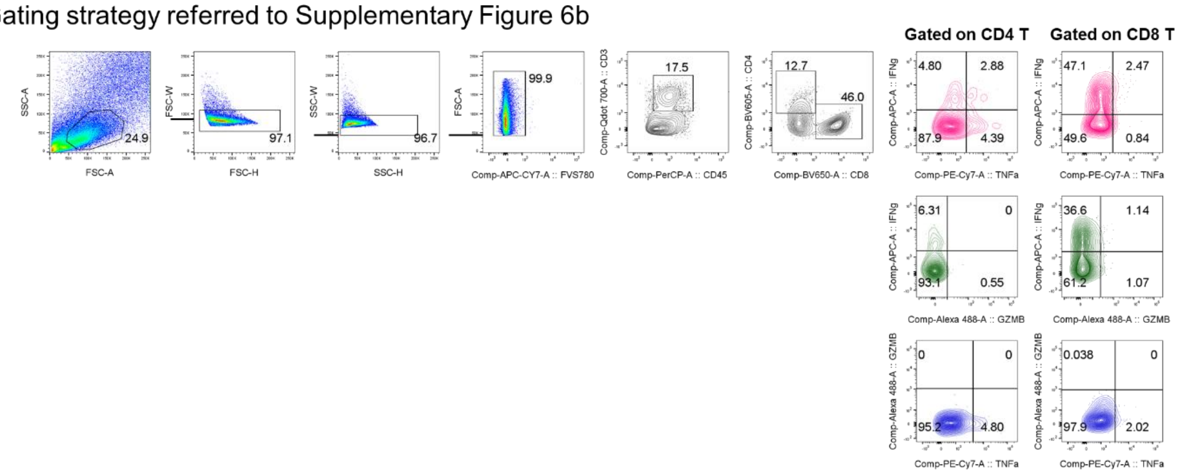

Supplementary Fig. 4 | Gating strategies for flow cytometric analyses of immune cells in Pan02 tumors. **a** Representative flow cytometry gating strategy for Ly6G^{+} Neutrophils, F4/80^{+}CD11b^{+} tumor-associated macrophages (TAM), CD19^{+} B cells, CD3^{+}CD4^{+} T cells, CD3^{+}CD8^{+} T cells. **b** Representative flow cytometry gating strategy for iNOS^{+} TAMs. **c** Representative flow cytometry gating strategy for IFN-γ^{+}TNF-α^{+}, IFN-γ^{+}TNF-α^{-}, IFN-γ^{-}TNF-α^{+} CD8^{+} T cells.

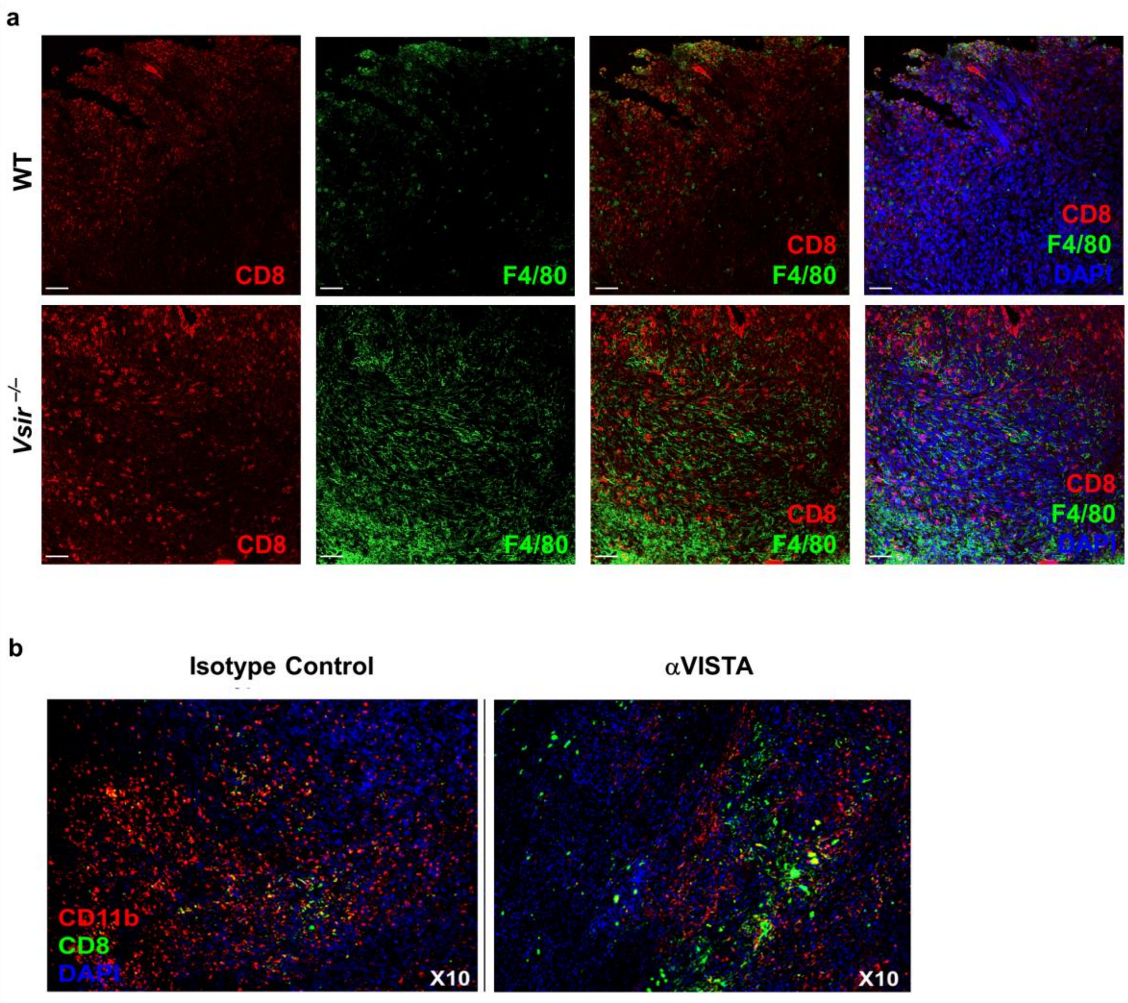

Supplementary Fig. 5 | CD11b+ myeloid and CD8+ T cells in WT *versus* anti-VISTA (αVISTA) antibody-treated tumor. a Representative immunofluorescence staining of CD8+ T cells (*red*) and F4/80+ Macrophages (*green*) in wild-type (WT) *versus Vsir*−/− tumors. Scale bar, 50 μm. **b** Representative immunofluorescence staining of CD11b+ myeloid cells (*red*) and CD8+ T cells (*green*) in tumors from isotype control *versus* αVISTA-treated mice. Images were captured at 10× magnification. Representative images from two independent experiments are shown.

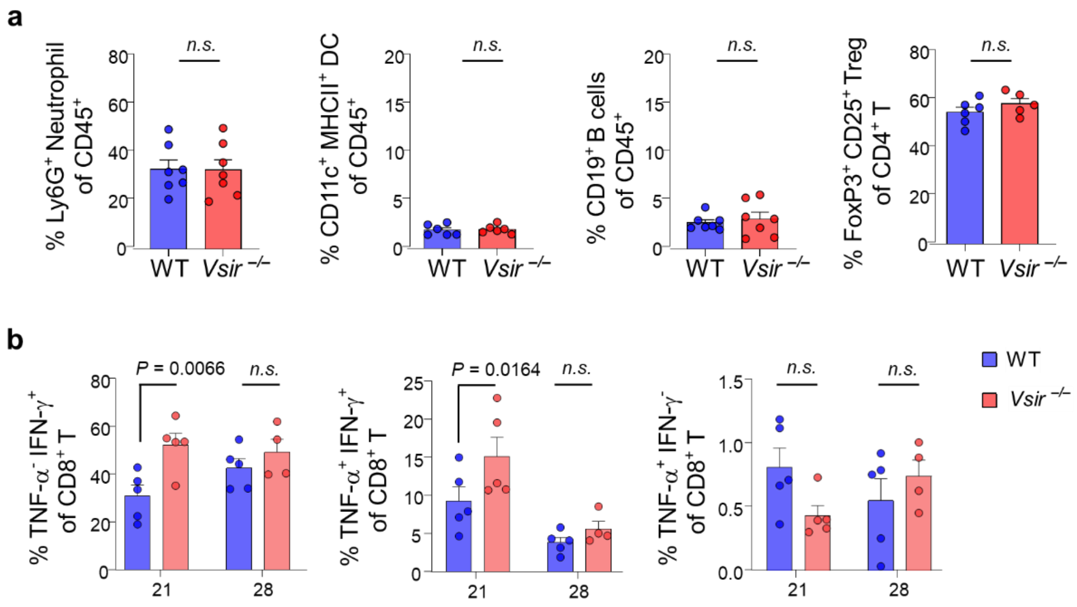

Supplementary Fig. 6 | Immune cell frequencies in Pan02 tumors. a Percentage of Ly6G$^+$ Neutrophils, CD11c$^+$MHCII$^+$ DCs, CD19$^+$ B cells, and FoxP3$^+$CD25$^+$ Treg cells within CD45$^+$ cells. (n = 6 per group). **b** Percentage of IFN-γ$^+$TNF-α$^+$, IFN-γ$^+$TNF-α$^-$, IFN-γ$^-$TNF-α$^+$ cells within CD8$^+$ T cells (WT, n = 10; VISTA KO, n = 9). Data are mean ± SEM. Statistical analysis was performed using an unpaired two-tailed Student's *t*-test for panels **a** and **b**. *Abbreviation:* WT, wild-type. *n.s.*, not significant.

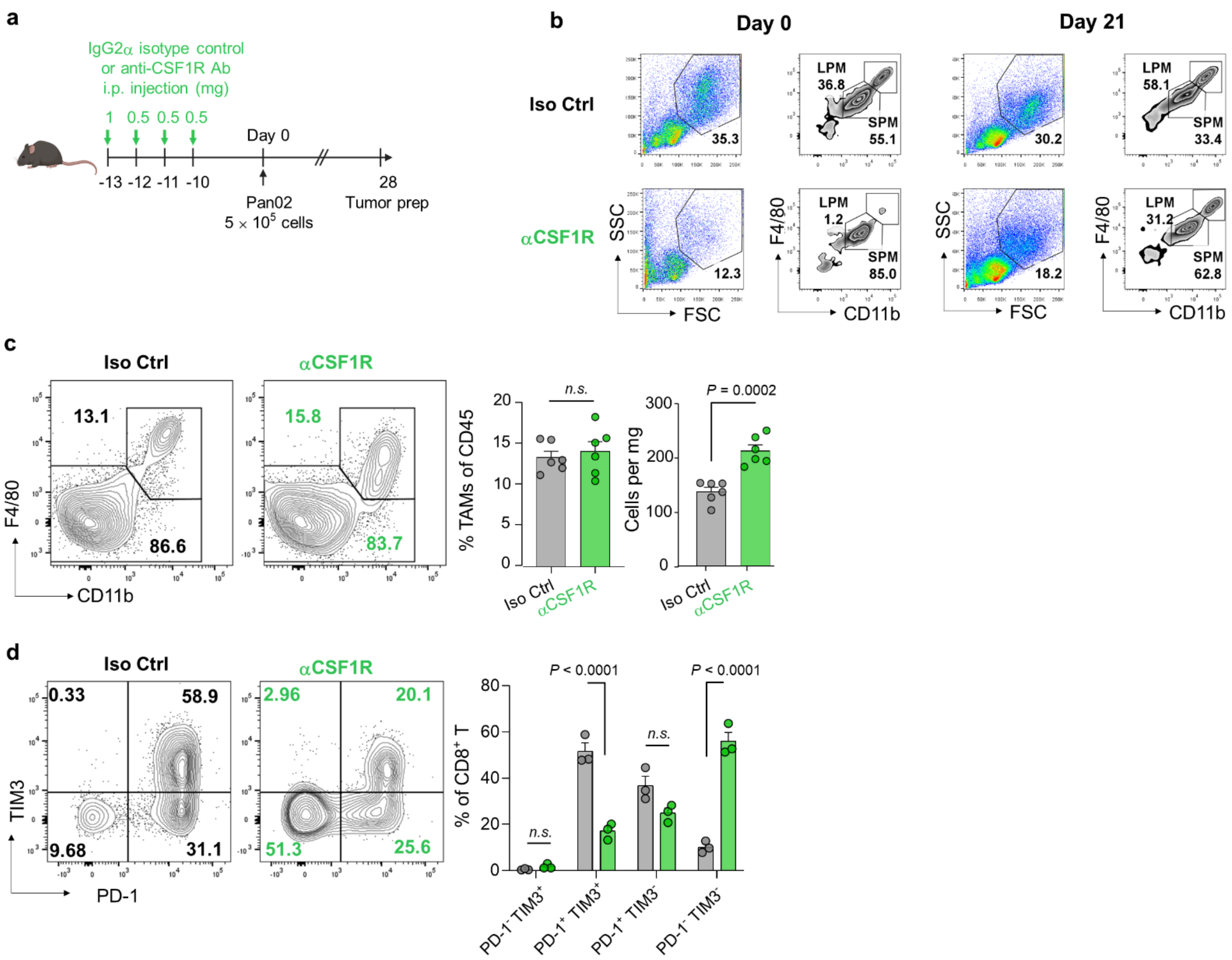

Supplementary Fig. 7 | Effect of anti-CSF1R (αCSF1R) antibody treatment. a Anti-CSF1R antibody treatment scheme. Created in BioRender. Kim, H. (2026) https://BioRender.com/ng9t0q2 **b** Representative flow cytometry plots showing effective depletion of F4/80^{high}CD11b^{+} tumor-associated macrophages (TAM) in wild-type (WT) mice treated with anti-CSF1R antibody on day 0 and day 21 after cancer cell inoculation. **c** Flow cytometric analysis of Gr-1^{-}F4/80^{+}CD11b^{+} TAMs in isotype control (Iso Ctrl) *versus* αCSF1R-treated WT mice. Quantification is shown as the percentage of CD45^{+} cells and normalized to tumor mass (n = 6 per group). **d** Flow cytometric analysis of PD-1 and TIM-3 expression on CD8^{+} T cells in isotype control *versus* αCSF1R-treated tumors. Quantification is shown as the percentage of CD8^{+} T cells (n = 6 per group). Data are presented as mean ± SEM and unpaired two-tailed Student's t-test was used for panels **c** and **d.** *n.s.*, not significant. All experiments were independently repeated at least twice; representative results are shown.

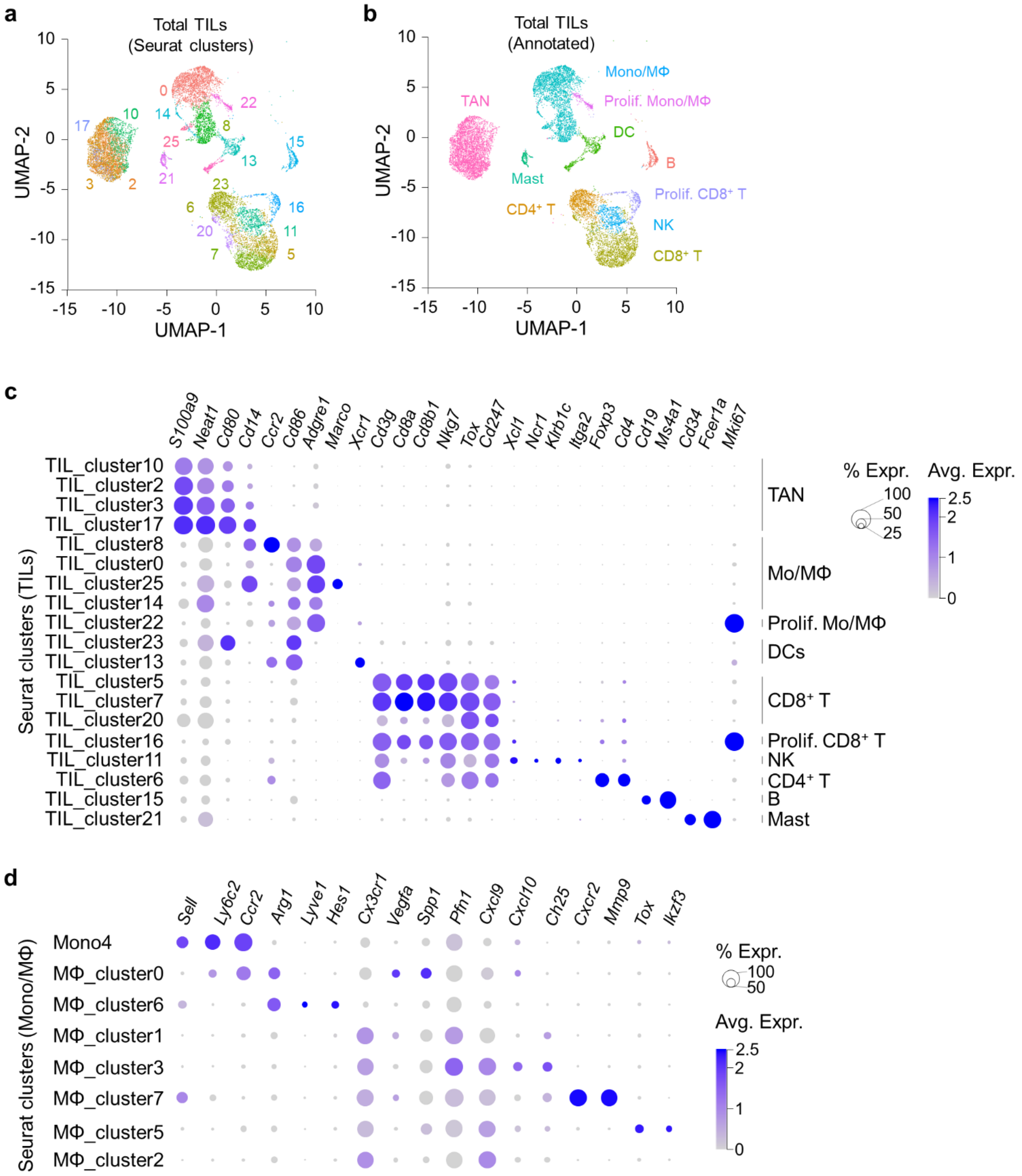

Supplementary Fig. 8 | UMAP of TILs from WT and *Vsir*$^{-/-}$ tumors and Mono/Macrophage subclusters. **a** Uniform manifold approximation and projection (UMAP) visualization of scRNA-seq data showing tumor-infiltrating lymphocytes (TILs) from wild-type (WT) and *Vsir*$^{-/-}$ tumors. Colors and numbers represent distinct transcriptional clusters. **b** UMAP showing annotated cell clusters of TILs from WT and *Vsir*$^{-/-}$ tumors. Colors correspond to specific immune cell type annotations. **c-d** Expression levels of marker genes used to identify TILs (**c**) and monocyte/macrophage subclusters (**d**).

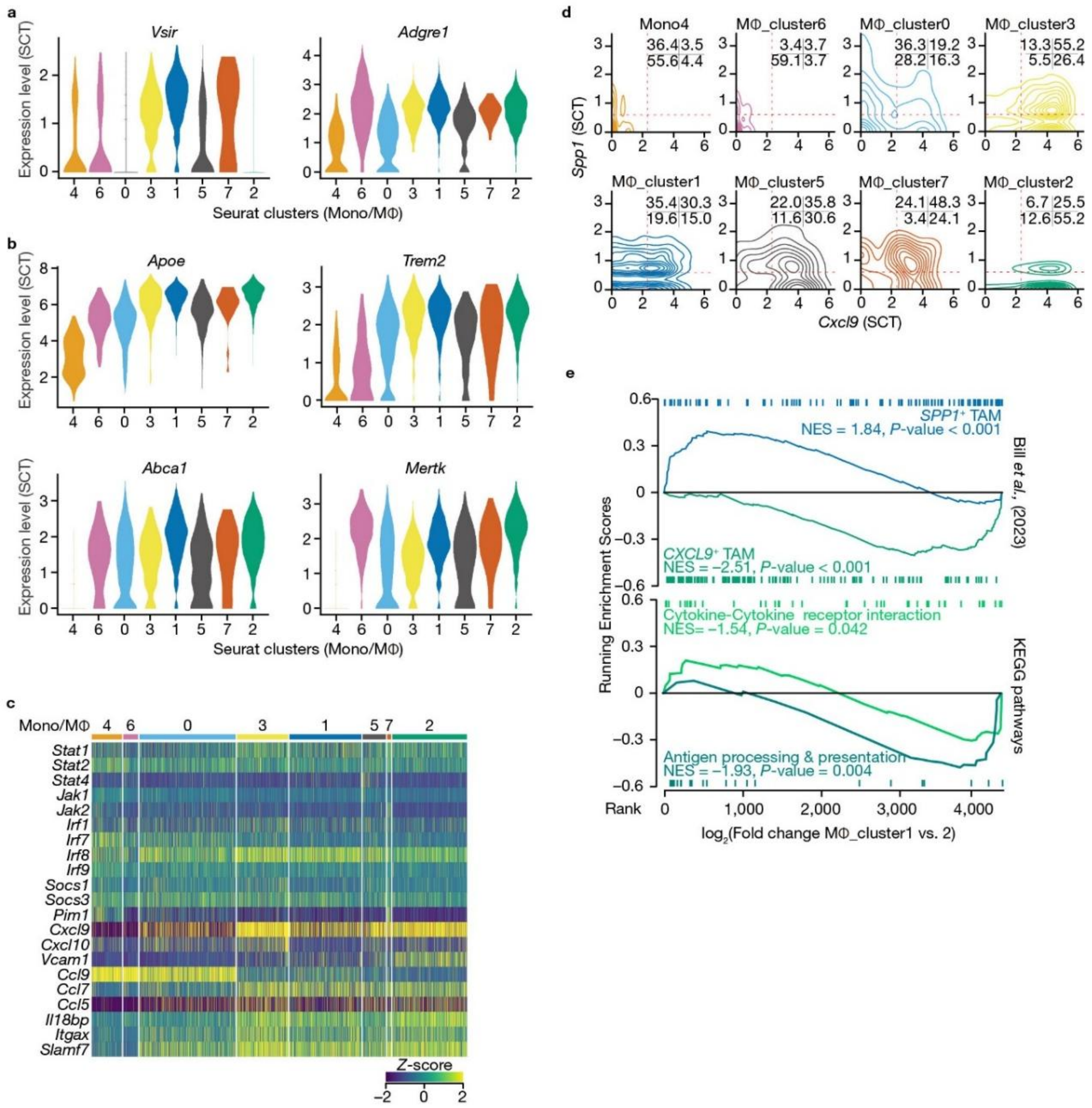

Supplementary Fig. 9 | Gene expression profiles from Mono/Macrophage subclusters. a SCT-normalized expression levels of *Vsir* and *Adgre1* in macrophage subclusters from wild-type (WT) and *Vsir*$^{-/-}$ mice. **b** SCT-normalized expression of *Apoe, Trem2, Abca1*, and *Mertk* in macrophage subclusters from WT and *Vsir*$^{-/-}$ mice. **c** Z-score-transformed gene expression profiles aligned across Mono/Macrophage clusters. **d** Density contour plots of macrophage clusters based on *Cxcl9* (*x*-axis) and *Spp1* (*y*-axis). Each plot represents two-dimensional kernel density estimation of single macrophages within a cluster. Dashed lines indicate the average expression level of each gene across all macrophages, defining quadrant thresholds. Cell frequencies within each quadrant are reported based on relative *Cxcl9* and *Spp1* expression. **e** Gene Set Enrichment Analysis (GSEA) of *Spp1*$^{+}$ and *Cxcl9*$^{+}$ tumor-associated macrophages (TAM) showing enrichment of pathways related to antigen processing and cytokine-cytokine receptor interaction. Normalized enrichment scores (NES) and corresponding permutation-derived *P* values are shown in the figure.

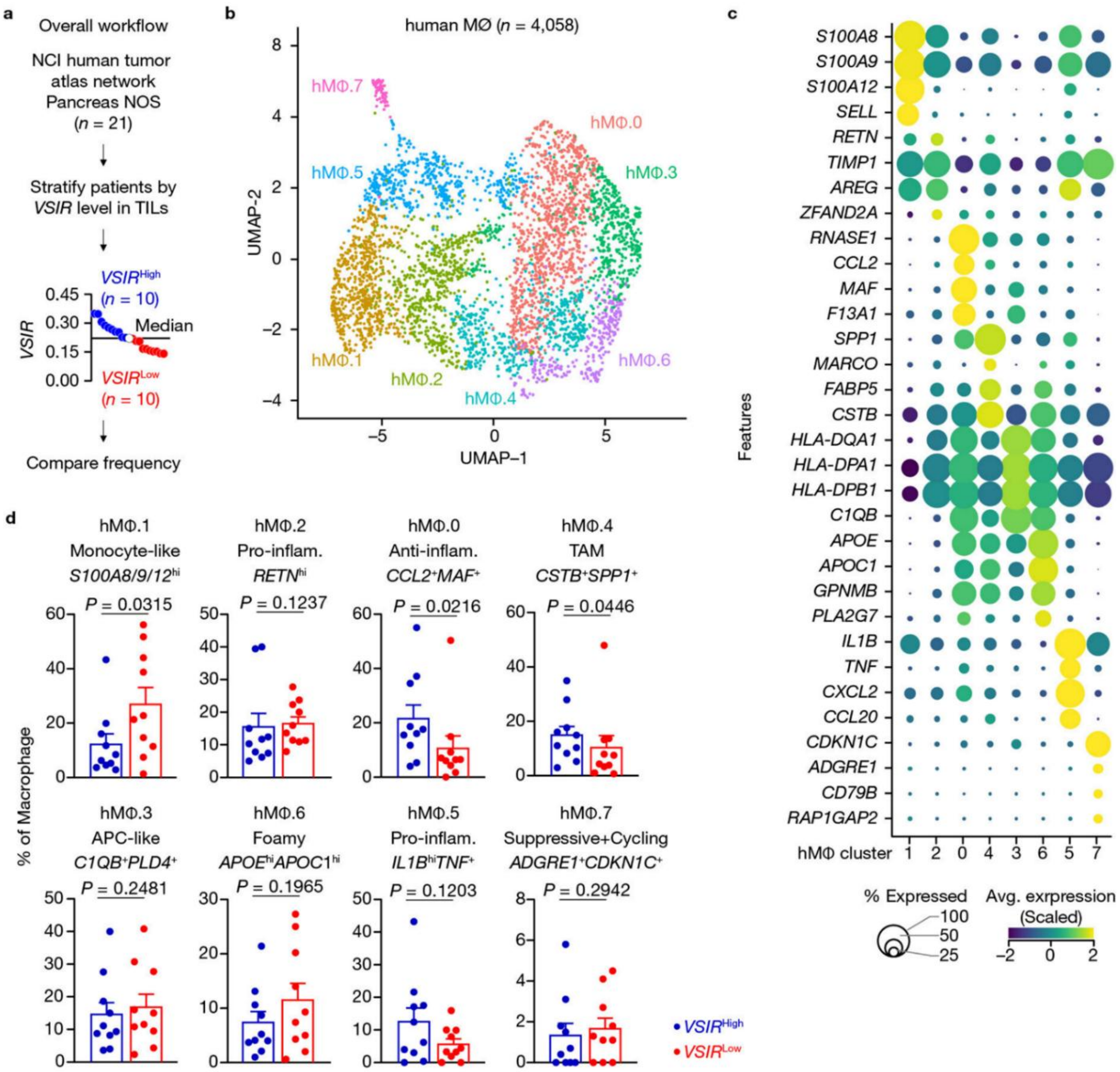

Supplementary Fig. 10 | Validation of *VSIR* expression in human scRNA-seq datasets. a Schematic workflow of human scRNA-seq analysis from the HTAN WUSTL cohort of pancreatobiliary-type carcinoma patients. **b** Uniform manifold approximation and projection (UMAP) visualization of macrophage subsets following subclustering. **c** Dot plot showing gene expression patterns across macrophage subsets. **d** Proportion of pro-inflammatory-like and anti-inflammatory-like genes in macrophages from *VSIR*^{high} (n = 10) *versus* *VSIR*^{low} (n = 10) patients. Patients were stratified by immune checkpoint expression: aggregate *VSIR* expression within the tumor-infiltrating lymphocytes (TIL) compartment was calculated for each patient, and the top and bottom 10 ranked patients were assigned to *VSIR*^{high} and *VSIR*^{low} groups, respectively. Group comparisons were performed using the one-sided Mann-Whitney U test. Exact P values are shown in the figures. *Abbreviation:* NCI, National Cancer Institute; NOS, Not Otherwise Specified.

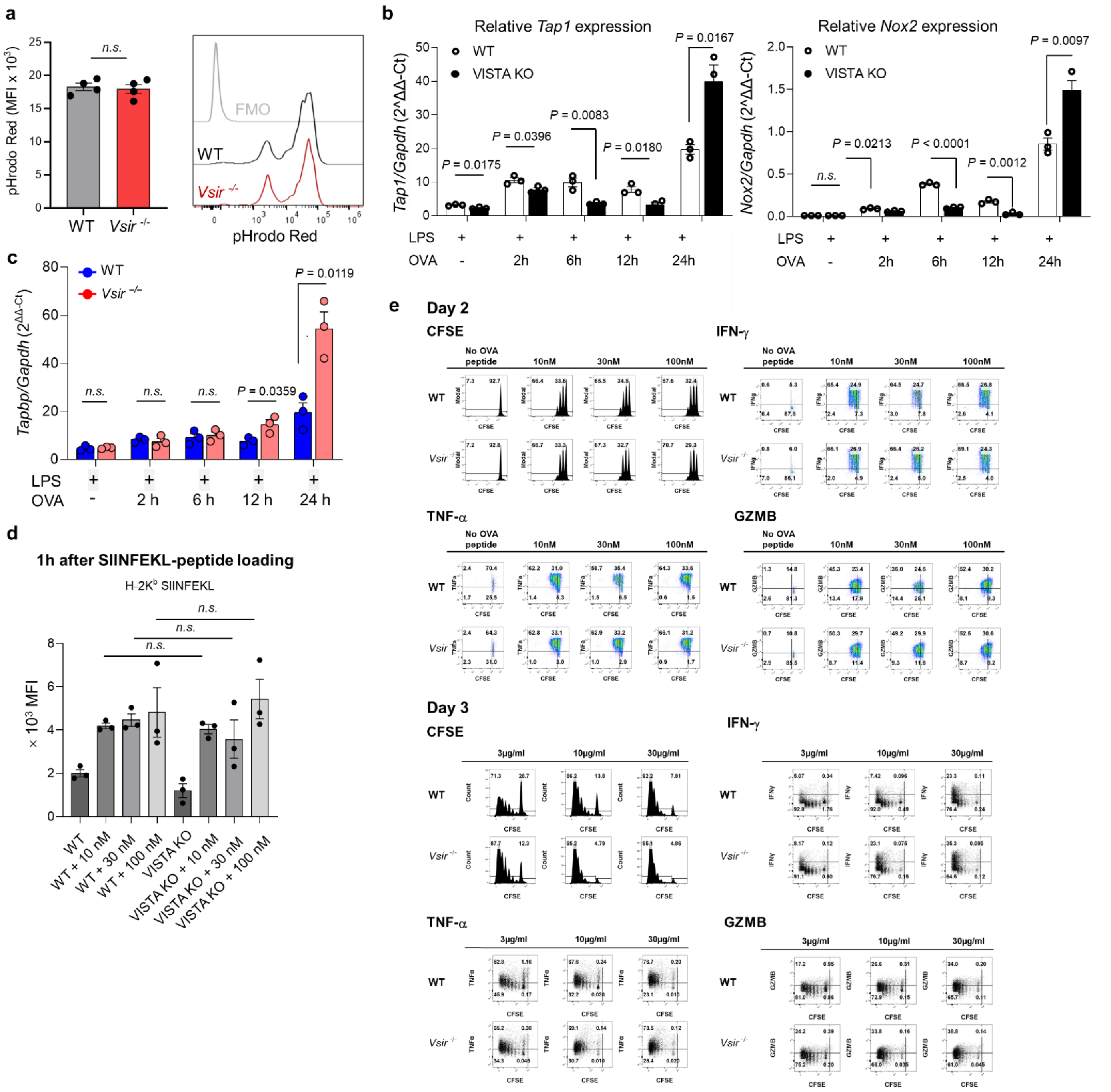

Supplementary Fig. 11 | *In vitro* characteristics of WT *versus* $Vsir^{-/-}$ BMDMs. a Mean fluorescence intensity (MFI; left) and representative histogram (right) of pHrodo™ Red zymosan bioparticle uptake by bone-marrow derived macrophages (BMDM) from wild-type (WT) and $Vsir^{-/-}$ mice (n = 3 biologically independent samples per group). **b** RT-qPCR analysis of *Tap1* and *Nox2* in WT and $Vsir^{-/-}$ BMDMs (n = 3 biologically independent samples per group). **c** *Tapbp* expression in WT and $Vsir^{-/-}$ BMDMs following lipopolysaccharide (LPS) plus ovalbumin (OVA) stimulation (n = 3 per group). **d** MFI of H-2K^b SIINFEKL complex on WT and $Vsir^{-/-}$ splenic dendritic cells (DC) (n = 3 per group). **e** Proliferation of carboxyfluorescein succinimidyl ester (CFSE)-labelled OT-I CD8$^+$ T cells co-cultured with WT or $Vsir^{-/-}$ splenic DCs loaded with

OVA, assessed on day 2 and day 3 of co-culture. All data are presented as mean ± SEM. Unpaired two-tailed Student's *t*-tests were applied to panels **a**–**d**. Exact *P* values are shown in the figures. *n.s.*, not significant. All experiments were independently repeated at least twice, and representative results are shown.

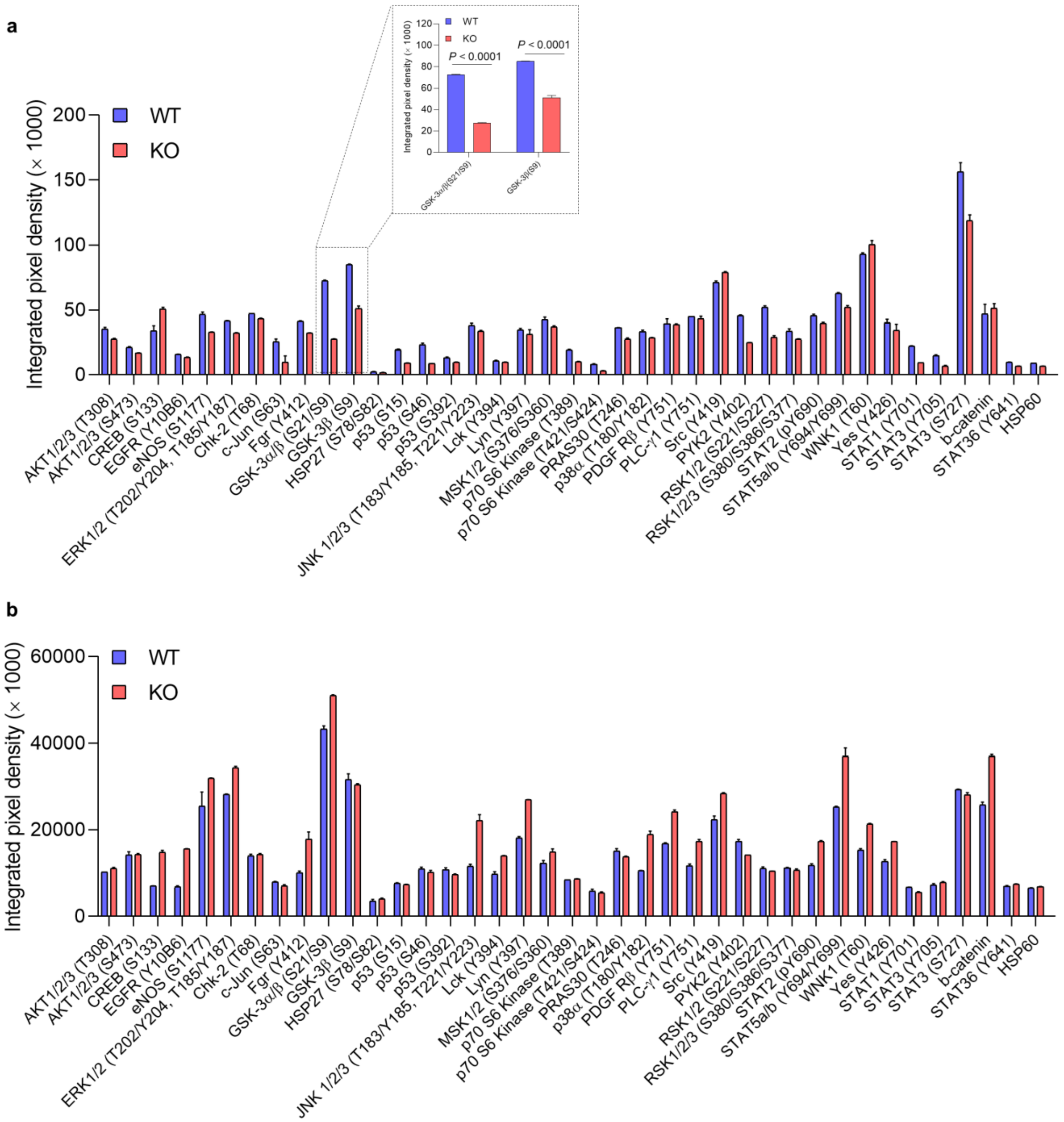

Supplementary Fig. 12 | Differential phosphoprotein signaling in wild-type (WT) and VISTA KO bone marrow-derived macrophages (BMDM) following inflammatory and immunoregulatory stimulation. **a** Integrated pixel density of phosphoproteins in WT and VISTA KO BMDMs following lipopolysaccharide (LPS) and IFN-γ stimulation. Quantification is shown alongside phospho-protein array membranes. **b** Integrated pixel density of phosphoproteins in WT and VISTA KO BMDMs following TGF-β1 and IL-10 stimulation. Data are presented from two biologically independent experiments with similar trends observed. Source data are provided as a Source Data file.

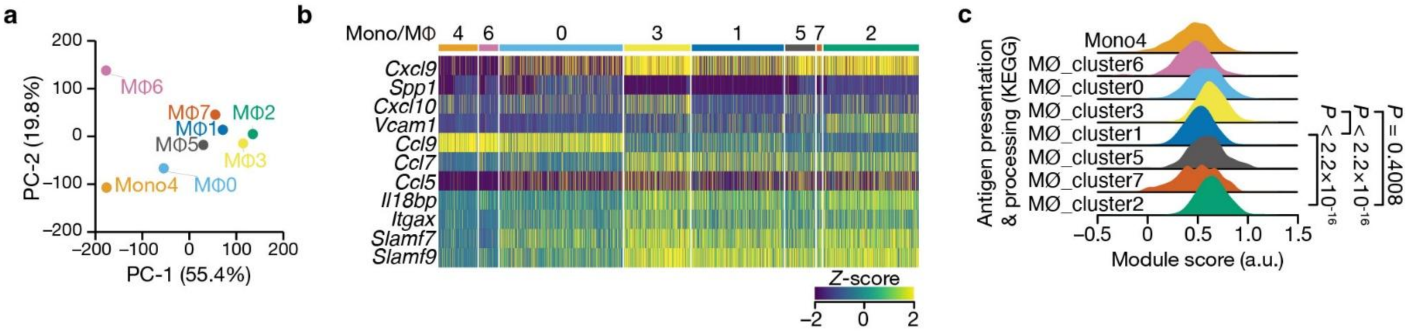

Supplementary Fig. 13 | Characterization of monocyte and macrophage clusters in the tumor microenvironment. a Uniform manifold approximation and projection (UMAP) visualization showing the distribution of macrophage and monocyte clusters. Clusters are labeled and color-coded as M*Φ*0 through M*Φ*7. **b** Z-score-transformed expression levels of chemokine-related genes across mono/macrophage subsets. **c** Module scores of the KEGG “Antigen processing and presentation” gene set calculated for monocyte/macrophage clusters. Statistical comparisons between clusters were performed using the two-sided Mann-Whitney *U* test. Exact *P* values are shown in the figure.

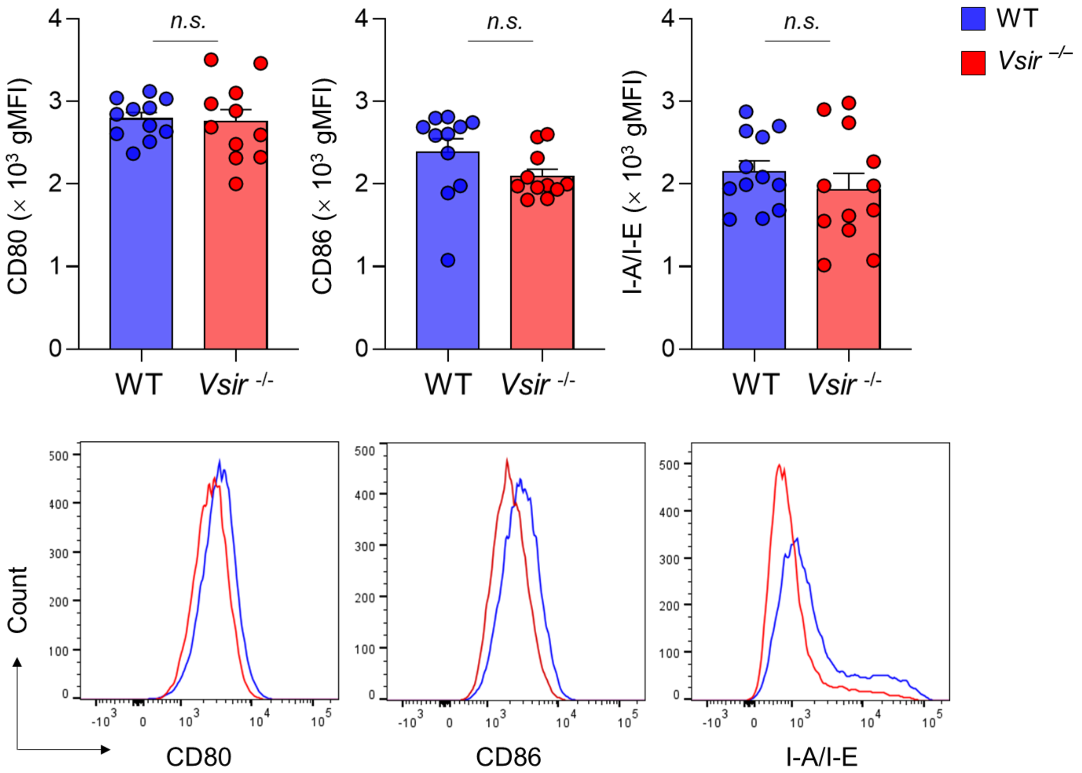

Supplementary Fig. 14 | Expression of CD80, CD86, and I-A/I-E in wild-type (WT) *versus Vsir*$^{-/-}$ bone marrow-derived macrophages (BMDM). Flow cytometric analysis of CD80 (n = 11 per group), CD86 (n = 11 per group), and I-A/I-E (n = 12 per group) expression on WT and *Vsir*$^{-/-}$ BMDMs. Quantification is shown as geometric mean fluorescence intensity (gMFI). Data are presented as mean ± SEM. Statistical significance was determined using an unpaired two-sided Student's t-test. *n.s.*, not significant.

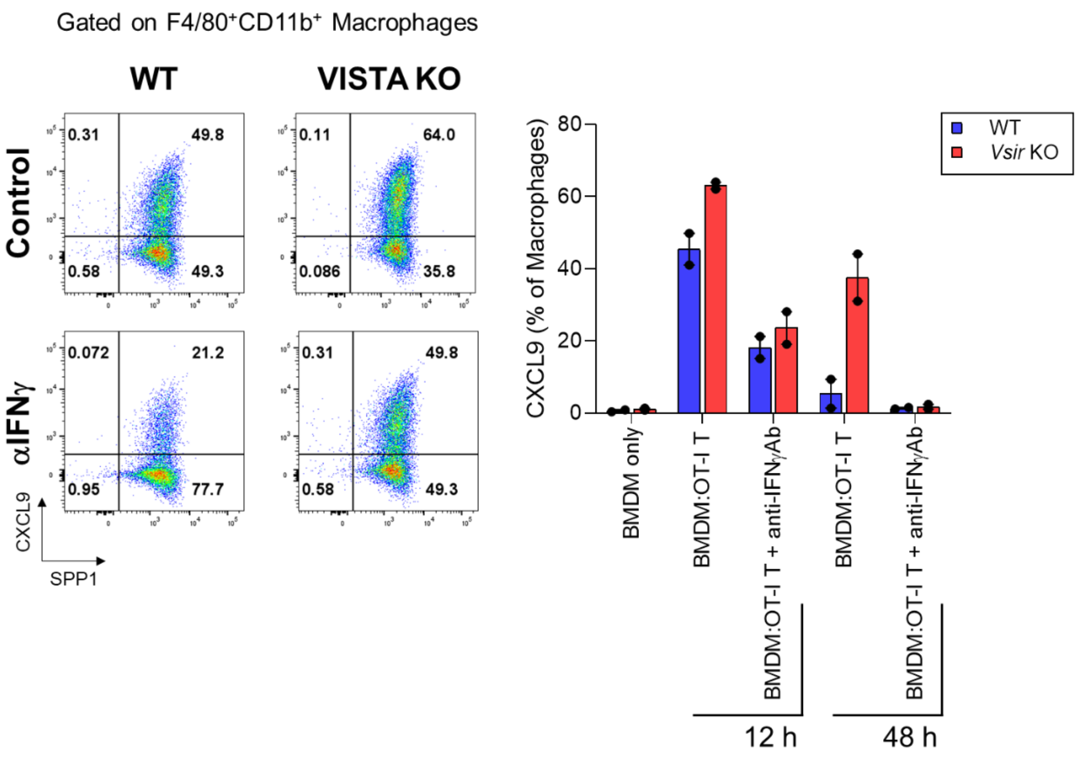

Supplementary Fig. 15 | Flow cytometric analysis of CXCL9⁺SPP1⁺ expression in wild-type (WT) and *Vsir*⁻/⁻ bone marrow-derived macrophages (BMDM) co-cultured with OT-I CD8⁺ T cells at a 1:1 ratio. Quantification is shown as the percentage of CXCL9⁺SPP1⁺ cells. Data are presented from two biologically independent experiments with similar trends observed. *Abbreviation:* αIFNγ, anti-IFN-gamma antibody.

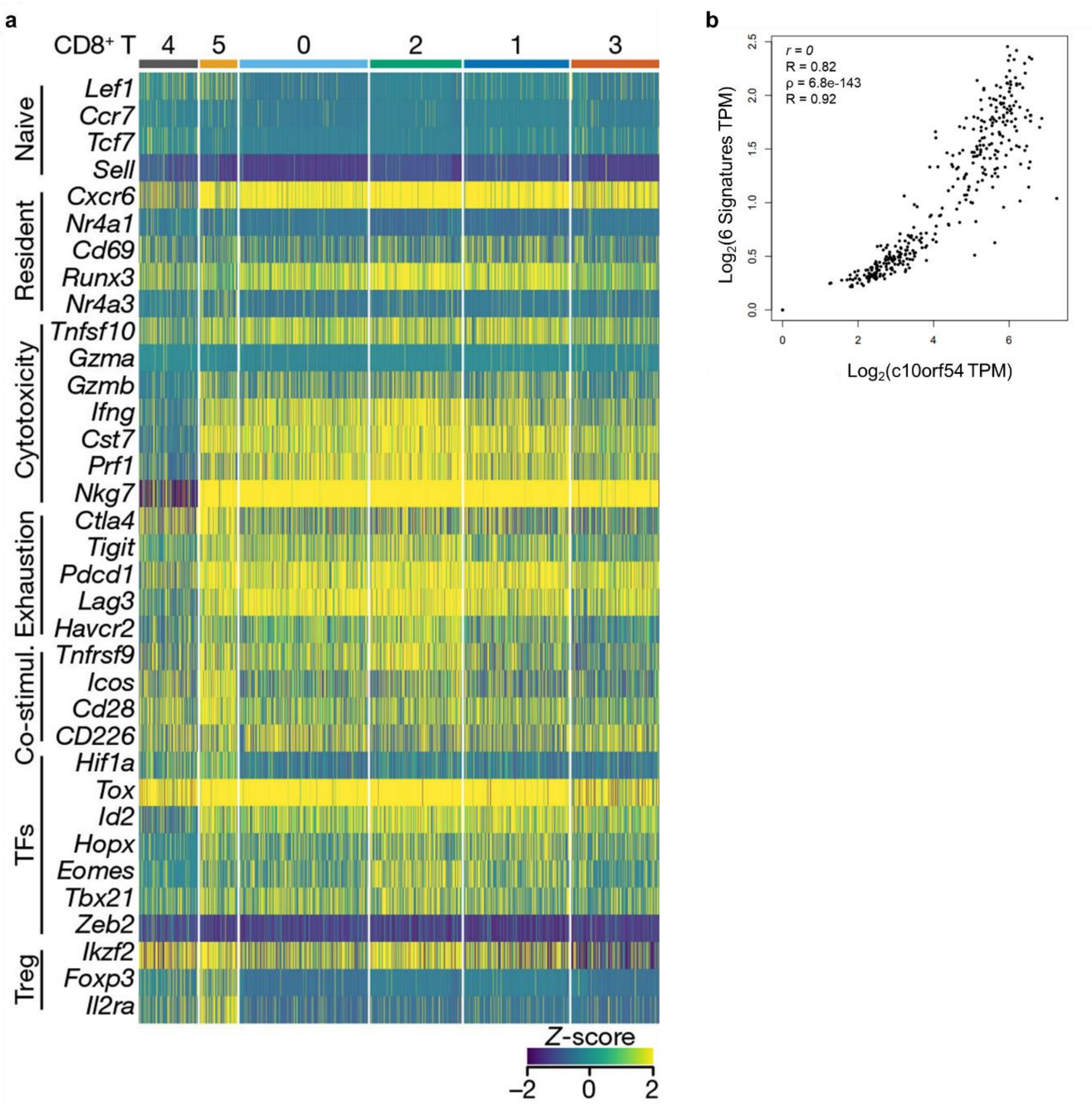

Supplementary Fig. 16 | Exhaustion-associated gene expression in CD8⁺ T cell subsets. a *Z*-score-transformed expression levels of gene sets related to naïve, resident, cytotoxic, exhaustion, co-stimulatory (Co-stimul.), transcription factors (TFs), and Treg-associated genes across CD8⁺ T cell subsets. **b** Correlation analysis between *Vsir* and six exhaustion markers (*Havcr2, Tigit, Lag3, Pdcd1, Cxcl13,* and *Layn)* in The Cancer Genome Atlas (TCGA) PAAD cohort (*n* = 179). Correlation analyses were performed using Pearson and Spearman tests (two-sided). Correlation coefficients (r and ρ) and exact *P* value are shown in the figure.

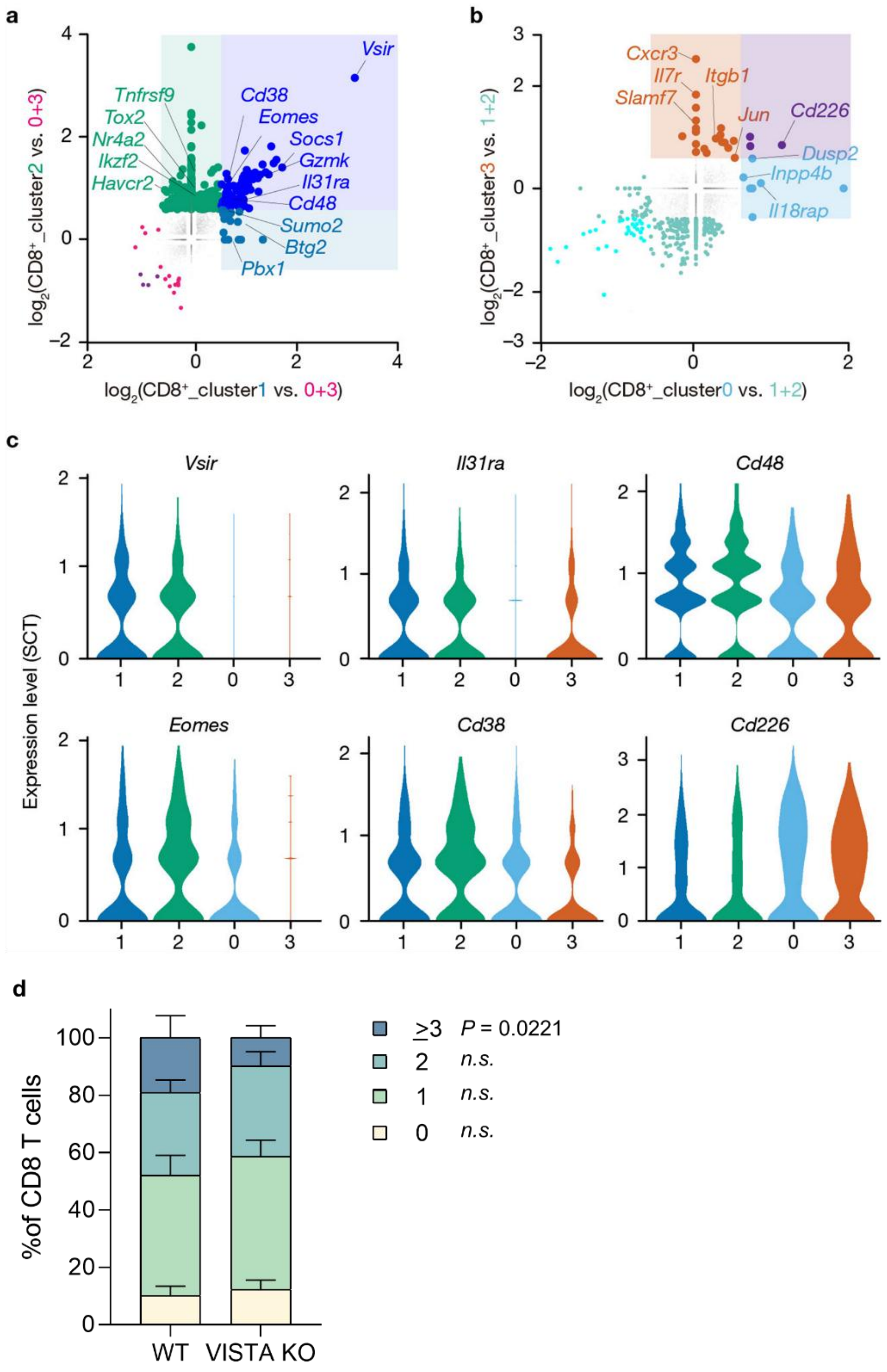

Supplementary Fig. 17 | Transcriptional differences between wild-type (WT)- and *Vsir*$^{-/-}$-specific CD8$^+$ T cell subclusters in the tumor microenvironment. a Scatter plot visualizing $\log_2$ fold changes in gene expression for CD8$^+$_cluster1 (*x*-axis) and CD8$^+$_cluster2 (*y*-axis), each compared with *Vsir*$^{-/-}$ clusters (CD8$^+$_cluster0 and CD8$^+$_cluster3), respectively (left). **b** Scatter plot visualizing $\log_2$ fold changes in gene expression for CD8$^+$_cluster0 (*x*-axis) and CD8$^+$_cluster3 (*y*-axis), each compared with WT clusters (CD8$^+$_cluster1 and CD8$^+$_cluster2), respectively. For panels **a** and **b**, each point represents a gene (as indicated in the figures). Source data are provided as a Source Data file. **c** Violin plots of normalized

expression levels of selected genes across CD8$^+$ T cell clusters. Clusters are grouped by genotype (WT-specific clusters 1 and 2;*Vsir*$^{-/-}$-specific cluster 0 and 3. **d** Frequency distribution of intratumoral CD8$^+$ T cells expressing 0, 1, 2, or ≥ 3 exhaustion markers (PD-1, TIM-3, LAG-3, and TIGIT) in KPC001 tumors (WT, *n* = 7; VISTA KO, *n* = 5). Data are presented as mean ± SEM. Statistical significance was determined using two-way ANOVA followed by Sidak's multiple-comparisons test (two-sided). Exact *P* values are shown in the figure.

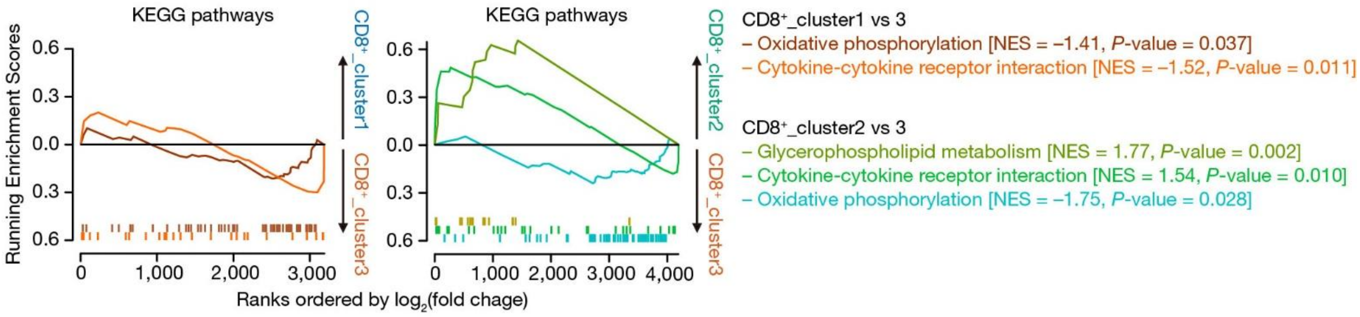

Supplementary Fig. 18 | Kyoto Encyclopedia of Genes and Genomes (KEGG) pathway enrichment in $CD8^+$ T cell clusters. Gene set enrichment analysis (GSEA) of KEGG pathways was performed for $CD8^+$ T cell clusters. The left panel shows pathways enriched in cluster 1 *versus* cluster 3, and the right panel shows pathways enriched in clusters 2 *versus* 3. Only gene sets with adjusted *P* values (*P*-value) < 0.05 are shown.

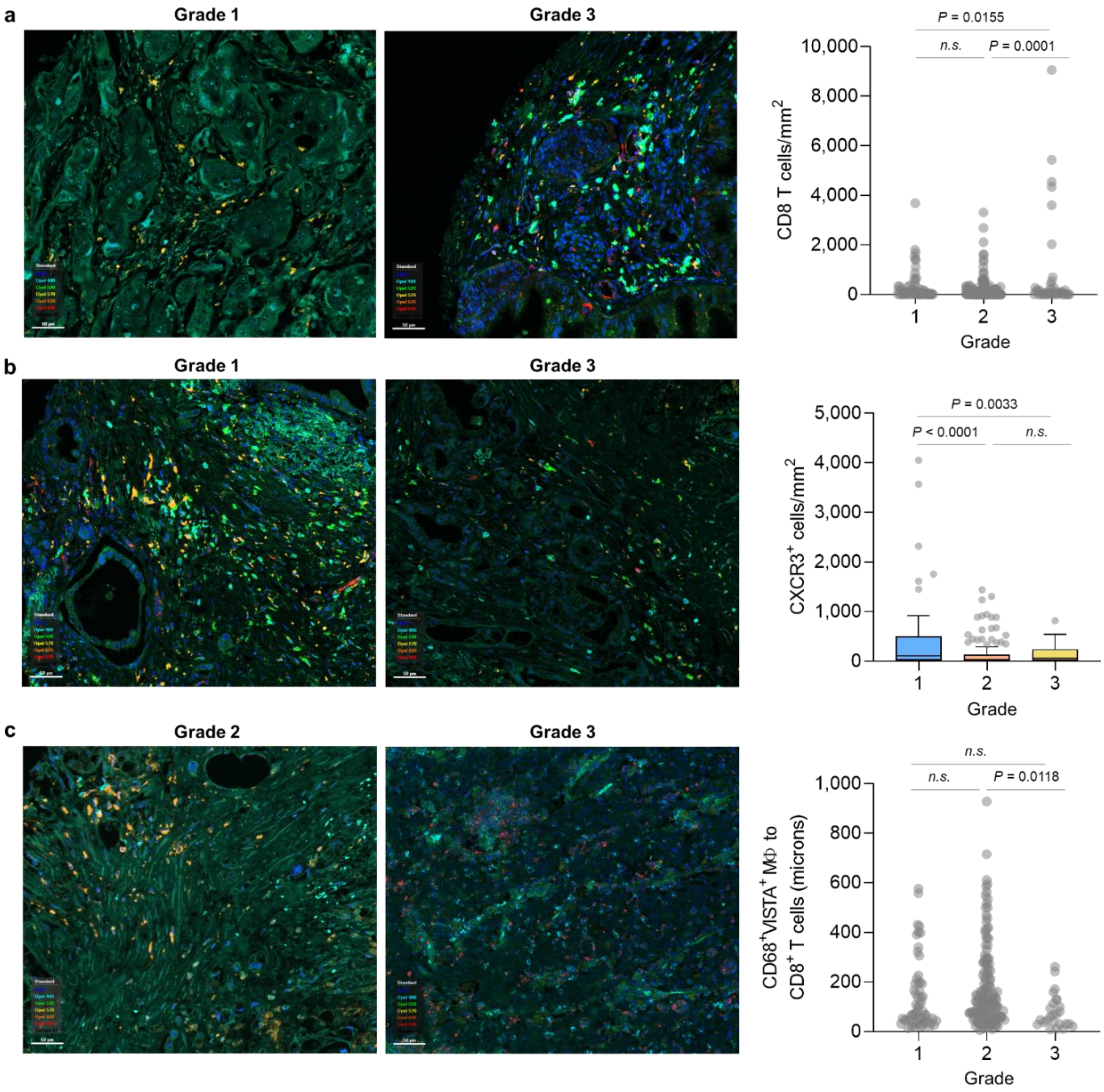

Supplementary Fig. 19 | **a** Representative images from grade 1 and grade 3 patient samples (20×; left) and quantification of $CD8^+$ T cell density (cells/mm²; right; Grade 1, *n* = 63; Grade 2, *n* = 183; Grade 3, *n* = 42). **b** Representative images from Grade 1 and Grade 3 patient samples (20×; left) and quantification of $CXCR3^+$ cell density (cells/mm²; right; Grade 1, *n* = 63; Grade 2, *n* = 183; Grade 3, *n* = 42). **c** Representative images from Grade 2 and Grade 3 patient samples (20×; left) and quantification of the nearest-neighbor distance between $CD68^+VISTA^+$ macrophages and $CD8^+$ T cells (right; Grade 1, *n* = 57; Grade 2, *n* = 152; Grade 3, *n* = 25). CD68, Opal 620; VISTA, Opal 690; CXCR3, Opal 520; and CD8α, Opal 480. Scale bar, 50 µm. Data are presented as box-and-whisker plot, along with gray circles representing outliers defined by Tukey's fence

method. Statistical significance was determined using one-way ANOVA followed by Tukey's multiple-comparisons test (two-sided). Exact *P* values are shown in the figures. *n.s.*, not significant.

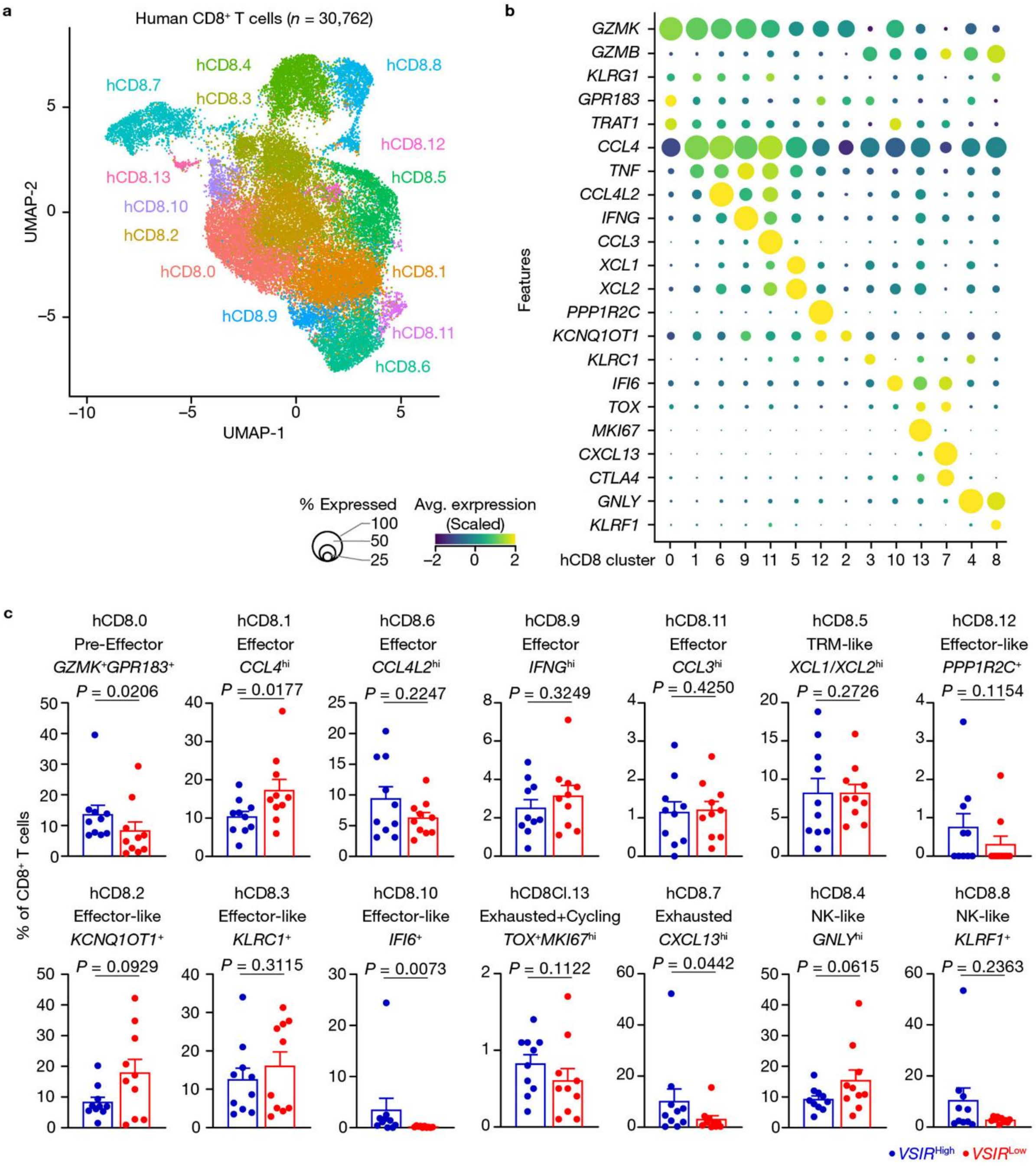

Supplementary Fig. 20 | Validation of *VSIR* expression in human scRNA-seq data. a Uniform Manifold Approximation and Projection (UMAP) visualization of $CD8^+$ T cell subsets following subclustering. **b** Dot plot showing gene expression profiles across identified $CD8^+$ T cell subsets. **c** Proportion of effector-like genes in *VSIR*^{high} *versus* *VSIR*^{low} patients. Patient groups were stratified using the same criteria as in Supplementary Fig. 10: aggregate *VSIR* expression within the TIL compartment was calculated for each patient from the HTAN WUSTL cohort, with the top and bottom 10 patients assigned to *VSIR*^{high} *versus* *VSIR*^{low} groups, respectively. Group comparisons were performed using the one-sided Mann-Whitney *U* test. Exact *P* values are shown in the figures.

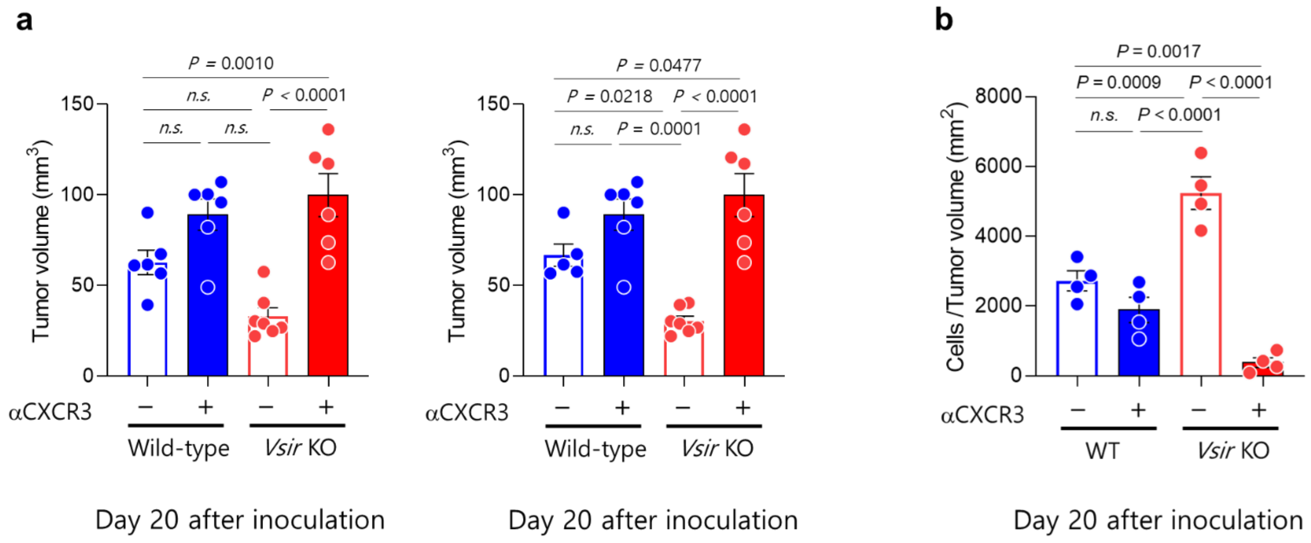

Supplementary Fig. 21 | Effect of CXCR3 blockade on tumor growth and $CD8^+$ T cell infiltration. a Tumor volume of wild-type (WT) and *Vsir* KO mice bearing Pan02 tumors on day 20 (n = 6 per group) and 27 (n = 5 per group) with or without anti-CXCR3 (αCXCR3) antibody administration. **b** Flow cytometric quantification of $CD8^+$ T cells normalized to tumor volume (n = 4 per group) at day 20 following αCXCR3 antibody administration. All data are presented as mean ± SEM. Statistical significance was determined using one-way ANOVA followed by Tukey's multiple-comparisons test (two-sided). Exact P values are shown in the figure. *n.s.*, not significant.

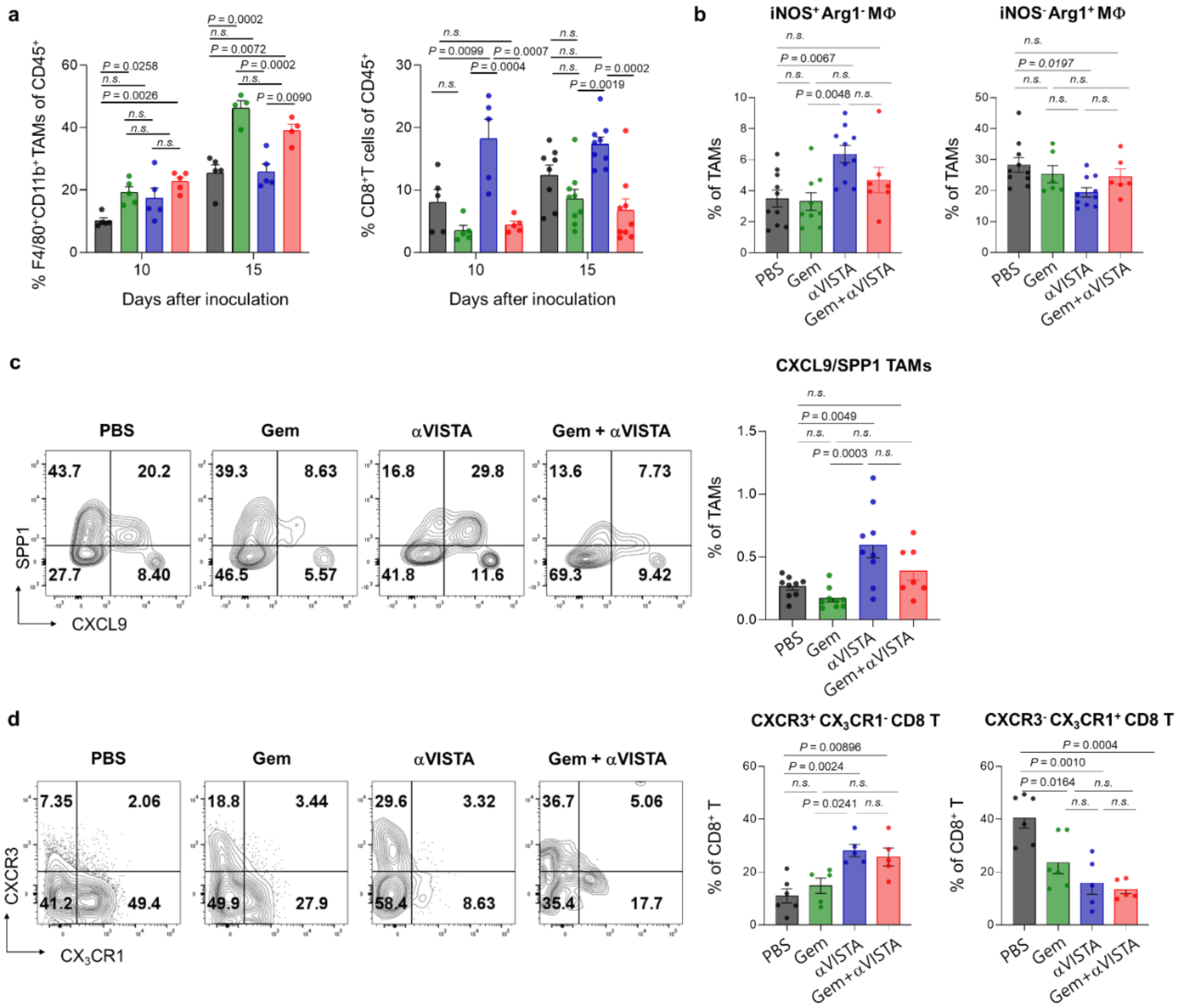

Supplementary Fig. 22 | Immune cell dynamics following combination therapy. a Flow cytometric quantification of F4/80$^+$CD11b$^+$ tumor-associated macrophages (TAM) (left; n = 5 per group at both time points) and CD8$^+$ T cells (right; n = 5 per group on day 10 and n = 9 per group on day 15) at days 10 and 15 post-treatment, expressed as a percentage of CD45$^+$ cells. **b** Flow cytometric analysis of iNOS$^+$Arg-1$^-$ (left) and iNOS$^-$Arg-1$^+$ (right) TAM subsets in tumors, shown as a percentage of CD45$^+$ cells (PBS and αVISTA, n = 10; Gem, n = 9; Gem + αVISTA, n = 7). **c** Representative flow cytometry plots (left) and quantification of CXCL9$^+$/SPP1$^+$ TAMs in tumors, expressed as a percentage of CD45$^+$ cells (PBS, Gem, and αVISTA, n = 9; Gem + αVISTA, n = 7). **d** Representative flow cytometry plots (left) and quantification of CXCR3$^+$CX$_3$CR1$^-$ CD8$^+$ T cells and CXCR3$^-$CX$_3$CR1$^+$ CD8$^+$ T cell subsets in tumors, shown as a percentage of CD45$^+$ cells (PBS, n = 6; Gem, αVISTA, and Gem + αVISTA, n = 5). All data are presented as mean ± SEM. Statistical

significance was determined using one-way ANOVA followed by Tukey's multiple-comparisons test (two-sided). Exact P values are shown in the figures. Experiments were independently repeated at least three times with similar results. *Abbreviation:* Gem, gemcitabine; αVISTA, anti-VISTA; *n.s.,* not significant.

Supplementary Table 1 | Multivariate Logistic Regression Analysis of Immune-Related Gene Associations with *VSIR* Expression (High *versus* Low), Adjusted for Age and Sex

	Odds ratio	95% Confidence Interval		*P*-value*
		Lower bound	Upper bound	
CD8B	0.0803	0.0343	0.1878	4.3174×10^{-9}
ADGRE1	0.1254	0.0567	0.2773	2.3155×10^{-7}
CD68	0.4612	0.2240	0.9495	0.0340
CCR2	0.0610	0.0257	0.1447	1.5407×10^{-10}
CX3CR1	0.2622	0.1265	0.5433	0.0003
CXCL9/SPP1	0.3893	0.1892	0.8009	0.0097

* Two-sided *P* values without adjustments for multiple-comparisons

Supplementary Table 2 | Oligonucleotide Primers for Real Time RT-qPCR Analysis

Gene	Direction	Sequence 5'-3'
Tapbp	Forward	CAGCTACCTCCAGTCACTGC
Tapbp	Reverse	GCCCTGAGAAGCCTGCCA
Gapdh	Forward	AGGTCGGTGTGAACGGATTTG
Gapdh	Reverse	GGGGTCGTTGATGGCAACA
Tap1	Forward	CTTGGATGATGCCACCAGTG
Tap1	Reverse	AGAAGAACCGTCCGAGAAGC
Nox2	Forward	AAGGCTTCAGGTCCACAGAGGAAA
Nox2	Reverse	AGACTTTGTATGGACGGCCCAACT

Supplementary Table 3 | Quantification of phospho-protein array results in WT and *Vsir* KO BMDMs under LPS and IFN-γ stimulation.

**LPS+IFN-γ**	WT		*Vsir* KO	
	Integrated pixel density (x1000)			
CREB (S133)	37.747	30.245	52.046	49.6
EGFR (Y10B6)	15.919	15.802	13.796	13.036
eNOS (S1177)	44.735	48.471	32.583	33.073
ERK1/2 (T202/Y204, T185/Y187)	41.247	41.886	32.467	32.024
Chk-2 (T68)	47.131	47.326	42.8	43.779
c-Jun (S63)	27.58	23.61	4.881	14.688
Fgr (Y412)	40.942	41.72	32.397	32.197
GSK-3α/β (S21/S9)	73	71.855	27.38	27.795
GSK-3β (S9)	85.278	84.551	53.053	49.035
HSP27 (S78/S82)	2.613	1.86	1.997	1.442
p53 (S15)	18.663	19.937	8.995	9.145
p53 (S46)	24.422	21.953	8.765	8.506
p53 (S392)	12.727	13.794	9.829	9.774
JNK 1/2/3 (T183/Y185, T221/Y223)	36.533	39.882	32.971	34.231
Lck (Y394)	11.313	10.261	9.876	9.752
Lyn (Y397)	33.393	35.721	27.944	34.68
MSK1/2 (S376/S360)	44.544	40.952	37.708	36.402
p70 S6 Kinase (T389)	18.496	19.772	10.084	10.317
p70 S6 Kinase (T421/S424)	8.59	7.823	3.274	2.904
PRAS30 (T246)	36.25	36.494	26.391	28.537
p38α (T180/Y182)	32.079	34.436	28.833	28.398
PDGF Rβ (Y751)	43.253	35.853	38.242	39.227
PLC-γ1 (Y751)	44.833	44.833	41.323	45.175
Src (Y419)	72.343	69.933	79.469	78.581
PYK2 (Y402)	45.148	46.2	24.768	24.859
RSK1/2 (S221/S227)	53.147	51.238	30.093	27.993
RSK1/2/3 (S380/S386/S377)	35.474	31.709	27.845	26.945
STAT2 (pY690)	46.826	44.661	40.411	38.97
STAT5a/b (Y694/Y699)	63.299	62.404	50.892	53.375
WNK1 (T60)	91.902	93.885	103.311	97.51
Yes (Y426)	42.941	37.474	38.968	29.426
STAT1 (Y701)	21.731	22.429	9.551	9.577
STAT3 (Y705)	15.467	14.417	5.882	7.373
STAT3 (S727)	163.35	149.139	114.539	123.152
β-catenin	54.361	39.305	54.789	47.764
STAT6 (Y641)	9.576	9.856	6.793	6.513
HSP60	8.986	8.985	6.615	6.89

Supplementary Table 4 | Quantification of phospho-protein array results in WT and *Vsir* KO BMDMs under TGF-β1 and IL-10 stimulation.

**TGF-β1+IL-10**	WT		*Vsir* KO	
	Integrated pixel density			
Chk-2 (T68)	13,593	14,372	14,512	14,065
c-Jun (S63)	7,901	8,067	6,771	7,323
Fgr (Y412)	10,487	9,781	19,488	16,305
GSK-3α/β (S21/S9)	42,564	43,982	50,767	51,219
GSK-3β (S9)	30,307	32,938	30,217	30,662
HSP27 (S78/S82)	3,952	3,215	3,957	4,214
p53 (S15)	7,516	7,772	7,413	7,211
p53 (S46)	10,599	11,403	9,775	10,650
p53 (S392)	10,448	11,240	9,490	9,816
JNK 1/2/3 (T183/Y185, T221/Y223)	11,109	12,048	23,483	20,880
Lck (Y394)	9,178	10,342	14,076	14,022
Lyn (Y397)	18,419	17,859	27,006	26,887
MSK1/2 (S376/S360)	11,760	12,888	14,200	15,575
p70 S6 Kinase (T389)	8,425	8,420	8,718	8,573
p70 S6 Kinase (T421/S424)	5,456	6,257	5,359	5,661
PRAS30 (T246)	14,699	15,629	13,602	13,903
p38α (T180/Y182)	10,648	10,512	19,728	18,015
PDGF Rβ (Y751)	16,650	16,995	24,575	23,813
PLC-γ1 (Y751)	11,384	12,092	16,866	17,791
Src (Y419)	21,501	23,207	28,560	28,186
PYK2 (Y402)	16,957	17,753	14,234	14,208
RSK1/2 (S221/S227)	10,722	11,428	10,420	10,493
RSK1/2/3 (S380/S386/S377)	11,319	11,116	10,332	10,983
STAT2 (pY690)	12,170	11,544	17,534	17,057
STAT5a/b (Y694/Y699)	25,163	25,454	35,065	38,943
WNK1 (T60)	15,655	15,001	21,174	21,461
Yes (Y426)	12,202	13,102	17,258	17,340
STAT1 (Y701)	6,778	6,616	5,697	5,553
STAT3 (Y705)	6,987	7,594	8,037	7,688
STAT3 (S727)	29,383	29,320	27,556	28,579
β-catenin	25,141	26,375	36,591	37,448
STAT6 (Y641)	6,848	7,148	7,352	7,529
HSP60	6,612	6,447	6,904	6,879
