# Supplementary material for: VISTA drives pancreatic tumor progression through modulation of the tumor-associated macrophage polarity
Source: Nat Commun. 2026 Mar 3;17:4582. doi: 10.1038/s41467-026-70215-7 (PMC13194722; doi:10.1038/s41467-026-70215-7)
Supplement: Supplementary file 2 — Reporting Summary [file 41467_2026_70215_MOESM2_ESM.pdf]

nature portfolio

Corresponding author(s): Hyun Mu Shin, Hang-Rae Kim, Dong-Sup Lee

Last updated by author(s): 02/22/2026

Reporting Summary

Nature Portfolio wishes to improve the reproducibility of the work that we publish. This form provides structure for consistency and transparency in reporting. For further information on Nature Portfolio policies, see our Editorial Policies and the Editorial Policy Checklist.

Statistics

For all statistical analyses, confirm that the following items are present in the figure legend, table legend, main text, or Methods section.

n/a	Confirmed	
☐	☒	The exact sample size (n) for each experimental group/condition, given as a discrete number and unit of measurement
☐	☒	A statement on whether measurements were taken from distinct samples or whether the same sample was measured repeatedly
☐	☒	The statistical test(s) used AND whether they are one- or two-sided<br>*Only common tests should be described solely by name; describe more complex techniques in the Methods section.*
☐	☒	A description of all covariates tested
☐	☒	A description of any assumptions or corrections, such as tests of normality and adjustment for multiple comparisons
☐	☒	A full description of the statistical parameters including central tendency (e.g. means) or other basic estimates (e.g. regression coefficient) AND variation (e.g. standard deviation) or associated estimates of uncertainty (e.g. confidence intervals)
☐	☒	For null hypothesis testing, the test statistic (e.g. F, t, r) with confidence intervals, effect sizes, degrees of freedom and P value noted<br>*Give P values as exact values whenever suitable.*
☒	☐	For Bayesian analysis, information on the choice of priors and Markov chain Monte Carlo settings
☒	☐	For hierarchical and complex designs, identification of the appropriate level for tests and full reporting of outcomes
☐	☒	Estimates of effect sizes (e.g. Cohen's d, Pearson's r), indicating how they were calculated

Our web collection on statistics for biologists contains articles on many of the points above.

Software and code

Policy information about availability of computer code

Data collection	TCGA-PAAD (TCGAbiolinks), HTAN WUSTL cohort (pancreatobiliary-type carcinoma)
Data analysis	RStudio (v3.18), (vSeurat (v3), Kaplan-Meier Plotter, Enrichr, IMARIS 9.3 software, Graph Prism (v8), ImageJ (v1.54), FlowJo (v10).

For manuscripts utilizing custom algorithms or software that are central to the research but not yet described in published literature, software must be made available to editors and reviewers. We strongly encourage code deposition in a community repository (e.g. GitHub). See the Nature Portfolio guidelines for submitting code & software for further information.

Data

Policy information about availability of data

All manuscripts must include a data availability statement. This statement should provide the following information, where applicable:

- Accession codes, unique identifiers, or web links for publicly available datasets
- A description of any restrictions on data availability
- For clinical datasets or third party data, please ensure that the statement adheres to our policy

The raw files of single-cell RNA sequencing data in this study have been deposited in the Gene Expression Omnibus database under accession code GSE282101 [https://www.ncbi.nlm.nih.gov/geo/query/acc.cgi?acc=GSE282101].

Research involving human participants, their data, or biological material

Policy information about studies with human participants or human data. See also policy information about sex, gender (identity/presentation), and sexual orientation and race, ethnicity and racism.

Reporting on sex and gender	*Use the terms sex (biological attribute) and gender (shaped by social and cultural circumstances) carefully in order to avoid confusing both terms. Indicate if findings apply to only one sex or gender; describe whether sex and gender were considered in study design; whether sex and/or gender was determined based on self-reporting or assigned and methods used.<br>Provide in the source data disaggregated sex and gender data, where this information has been collected, and if consent has been obtained for sharing of individual-level data; provide overall numbers in this Reporting Summary. Please state if this information has not been collected.<br>Report sex- and gender-based analyses where performed, justify reasons for lack of sex- and gender-based analysis.*
Reporting on race, ethnicity, or other socially relevant groupings	*Please specify the socially constructed or socially relevant categorization variable(s) used in your manuscript and explain why they were used. Please note that such variables should not be used as proxies for other socially constructed/relevant variables (for example, race or ethnicity should not be used as a proxy for socioeconomic status).<br>Provide clear definitions of the relevant terms used, how they were provided (by the participants/respondents, the researchers, or third parties), and the method(s) used to classify people into the different categories (e.g. self-report, census or administrative data, social media data, etc.)<br>Please provide details about how you controlled for confounding variables in your analyses.*
Population characteristics	*Describe the covariate-relevant population characteristics of the human research participants (e.g. age, genotypic information, past and current diagnosis and treatment categories). If you filled out the behavioural & social sciences study design questions and have nothing to add here, write "See above."*
Recruitment	*Describe how participants were recruited. Outline any potential self-selection bias or other biases that may be present and how these are likely to impact results.*
Ethics oversight	*Identify the organization(s) that approved the study protocol.*

Note that full information on the approval of the study protocol must also be provided in the manuscript.

Field-specific reporting

Please select the one below that is the best fit for your research. If you are not sure, read the appropriate sections before making your selection.

☒ Life sciences ☐ Behavioural & social sciences ☐ Ecological, evolutionary & environmental sciences

For a reference copy of the document with all sections, see nature.com/documents/nr-reporting-summary-flat.pdf

Life sciences study design

All studies must disclose on these points even when the disclosure is negative.

Sample size	For animal assay, sample sizes were selected empirically from previous experimental experience with similar assays, and/or from sizes generally employed in the field.<br>For cell assay, sizes were selected empirically from previous experimental experience with similar assays.
Data exclusions	No data was excluded.
Replication	Data are representative of at least 2 independent experiments, and all attempts at replication were successful.
Randomization	For animal assay, seven to nine weeks-old male mice were randomly assigned to different treatment groups.
Blinding	The investigators were blinded to group allocation during data collection and/or analysis.

Reporting for specific materials, systems and methods

We require information from authors about some types of materials, experimental systems and methods used in many studies. Here, indicate whether each material, system or method listed is relevant to your study. If you are not sure if a list item applies to your research, read the appropriate section before selecting a response.

Materials & experimental systems

n/a	Involved in the study
	☒ Antibodies
	☒ Eukaryotic cell lines
☒	Palaeontology and archaeology
	☒ Animals and other organisms
☒	Clinical data
☒	Dual use research of concern
☒	Plants

Methods

n/a	Involved in the study
☒	ChIP-seq
	☒ Flow cytometry
☒	MRI-based neuroimaging

Antibodies

Antibodies used

(1) In vivo treatments
For in vivo experiments, the following antibodies were used: Anti-VISTA antibody (Clone 13F3, InVivoMab, BioXCell, Lebanon, NH, USA), Armenian Hamster IgG isotype control (Polyclonal, InVivoMab, BioXCell), Anti-CS1FR antibody (Clone AFS98, InVivoMab, BioXCell), Rat IgG2alpha,kappa isotype control (Clone 2A3, InVivoMab, BioXCell), Anti-IFN-gamma neutralizing antibody (Clone XMG1.2, InVivoMab, BioXCell), Anti-CXCR3 neutralizing antibody (Clone CXCR3-173, InVivoMab, BioXCell).

(2) Flow cytometry
For surface staining, the following antibodies were used: CD3 (BV711, 17A2, BioLegend; 1:500), CD4 (BV605, GK1.5, BioLegend; 1:500), CD4 (FITC, RM45, BD Pharmingen; 1:1000), CD8 (BV650, 53-6.7, BioLegend; 1:500), CD8 (APC, 53-6.7, BioLegend; 1:1000), CD45.2 (PerCPCy5.5, 104, BioLegend; 1:500), I-A/I-E (BV605, M5/11, BD Horizon; 1:500), F4/80 (BV711, T45-2342, BD Horizon; 1:500), CD11b (BV785, M1/70, BioLegend; 1:3000), CD11c (APC-Cy7, N418, BioLegend; 1:1000), Ly6G (APC, 1A8-Ly6g, Invitrogen; 1:500), Ly6C (PE-Cy7, HK1.4, BioLegend; 1:500), Gr-1 (BV510, RB6-8C5, BD Horizon; 1:1000), CD62L (BV605, MEL-14, BioLegend; 1:1000), CD44 (BV510, IM7, BioLegend; 1:500), TIM3 (PE, B8.2C12, BioLegend; 1:500), PD-1 (FITC, 29F.1A12, BioLegend; 1:100), VISTA (APC, MIH63, BioLegend; 1:200), CX3CR1 (Pacific Blue, SA011F11, BioLegend; 1:500), CXCR3 (BV421, CXCR3-173, BioLegend; 1:200), CCR2 (BV421, SA203G11, BioLegend; 1:400), CD206 (BV650, C068C2, BioLegend; 1:500), CD80 (PE, 16-10A1, BioLegend; 1:500), CD86 (FITC, GL-1, BioLegend; 1:500).
For intracellular staining, the following antibodies were used: CXCL9 (PE, MIG-2F5.5, BioLegend; 1:200), OPN (AlexaFluor647, LFMb-14, SantaCruz; 1:200), IFN-γ (APC, XMG1.2, BioLegend; 1:500), TNF-α (PE, Mab11, BioLegend; 1:500), Granzyme B (FITC, GB11, BioLegend; 1:500), and Perforin (PE, S16009A, BioLegend; 1:500).

(3) Immunohistochemistry (IHC)
For IHC, the following primary antibodies were used: CD3 (SP162, Abcam, Cambridge, UK; 1:300), CD8 (EPR21916, Abcam; 1:1000), CD11b (EPR1344, Abcam; 1:4000), F4/80 (EPR26545-166, Abcam; 1:5000), and anti-VISTA (D1L2G, Cell Signaling Technology, Danvers, MA, USA). Secondary antibodies: Goat anti-rabbit IgG H&L (Biotin, Abcam; 1:500), Goat anti-Rat IgG H&L (Biotin, Abcam; 1:500).
For IF, the following primary antibodies were used: CD8 (4SM15, Novus Biologicals, Centennial, Co, USA; 1:300), F4/80 (A3-1, Abcam; 5 ug/mL) and CD11b (EPR1344, Abcam; 1:500). The secondary antibodies: Goat anti-rat IgG H&L AF488 (Thermo Fisher Scientific, Waltham, MA, USA; 1:500), goat anti-rabbit IgG H&L AF647 (Thermo Fisher Scientific; 1:500), goat anti-rat IgG H&L AF488 (Thermo Fisher Scientific; 1:500), and goat anti-rabbit IgG H&L AF647 (Thermo Fisher Scientific; 1:500).

(4) Opal multiplex IF: Anti-VISTA (D1L2G, Cell Signaling Technology, Danvers, MA, USA), CD68 (EPR20545, Abcam), CXCR3 (EPR25373-32, Abcam), CD8alpha (EPR22483-288, Abcam), CD68-Opal 620, VISTA-Opal 690, CXCR3-Opal 520, CD8a-Opal 480.

Validation

Signals of all the fluorescence-conjugated antibodies were validated via fluorescence-minus-one (FMO) controls and/or IgG controls with mouse tumor and spleen tissues.

Eukaryotic cell lines

Policy information about cell lines and Sex and Gender in Research

Cell line source(s)

Pan02 cell line and KPC001 cell line (KrasG12D p53R172H/+) were kindly provided by Dr. Yves Boucher (Massachusetts General Hospital, Boston). Pan02-OVA cell line was made from Pan02 cell line according to Methods section.

Authentication

None of the cell lines were manually authenticated.

Mycoplasma contamination

All cells used in this study were tested for mycoplasma contamination.

Commonly misidentified lines
(See ICLAC register)

No commonly misidentified line was used in this study.

Animals and other research organisms

Policy information about studies involving animals; ARRIVE guidelines recommended for reporting animal research, and Sex and Gender in Research

Laboratory animals	Experiments were performed on C57BL/6J mice, 7-9 weeks, and mice were kept under specific pathogen-free conditions with free access to food and water and mice were maintained in 12 hour light/dark cycle with 23 °C ambient temperature and 40-60% humidity.
Wild animals	This study did not involve wild animals.
Reporting on sex	No sex analysis was performed.
Field-collected samples	All mice were housed in a ventillated temperature-controlled environment (median temperature 23 °C, humidity 40-60%), under a 12-hour light/dark cycle, with a free access to food and water. Mouse cages were changed weekly. No animals were excluded.
Ethics oversight	All experimental protocols were approved by the Institutional Animal Care and Use Committee (IACUC) of Seoul National University (No. SNU-220222-8-11).

Note that full information on the approval of the study protocol must also be provided in the manuscript.

Plants

Seed stocks	*Report on the source of all seed stocks or other plant material used. If applicable, state the seed stock centre and catalogue number. If plant specimens were collected from the field, describe the collection location, date and sampling procedures.*
Novel plant genotypes	*Describe the methods by which all novel plant genotypes were produced. This includes those generated by transgenic approaches, gene editing, chemical/radiation-based mutagenesis and hybridization. For transgenic lines, describe the transformation method, the number of independent lines analyzed and the generation upon which experiments were performed. For gene-edited lines, describe the editor used, the endogenous sequence targeted for editing, the targeting guide RNA sequence (if applicable) and how the editor was applied.*
Authentication	*Describe any authentication procedures for each seed stock used or novel genotype generated. Describe any experiments used to assess the effect of a mutation and, where applicable, how potential secondary effects (e.g. second site T-DNA insertions, mosiacism, off-target gene editing) were examined.*

Flow Cytometry

Plots

Confirm that:

☒ The axis labels state the marker and fluorochrome used (e.g. CD4-FITC).

☒ The axis scales are clearly visible. Include numbers along axes only for bottom left plot of group (a 'group' is an analysis of identical markers).

☒ All plots are contour plots with outliers or pseudocolor plots.

☒ A numerical value for number of cells or percentage (with statistics) is provided.

Methodology

Sample preparation

(1) Tumor dissociation
Mouse pancreatic tumors were manually minced in small pieces. Tissues were suspended in RPMI-1640 supplemented with DNase I (1 mg/mL, Roche, Basel, Switzerland) and Collagenase IV (1 mg/mL, Roche) and incubated at 37°C for 20 min, with frequent agitation. The digested cell suspensions were then filtered through a 70-um nylon cell strainer and treated with 1x RBC lysis buffer (BioLegend, San Diego, CA, USA) for 15 min on ice and resuspended in the appropriate buffer for cell counting and downstream application.

(2) Splenic dendritic cells
Spleens were harvested from WT or Vsir–/– mice, minced into small pieces, and agitated in Hank's Balanced Salt Solution (HBSS) containing Collagenase IV (1 mg/mL, Roche), DNase I (50 ug/mL, Roche), and 1% FBS for 20 min at 37oC. The enzymatic reaction was stopped by adding 1 mM EDTA for 5 min at RT. The cell suspension was filtered through a 70-um cell strainer. CD11c+ DCs were enriched using magnetic-activated cell sorting (MACS Microbeads and Separation Unit, Miltenyi Biotec, Bergisch Gladbach, Germany).

(3) Bone marrow derived macrophages
Bone marrow was flushed from the femurs and tibias of either WT or Vsir–/– mice, filtered, and red blood cells were lysed. Cells were washed and plated at 2 x 10^6 cells/mL in non-treated six-well plates containing DMEM (Gibco) supplemented with 10% FBS, 1% Antibiotic/Antimycotic, and 10% L929 supernatant. On day 3, cells were refed with fresh media. At day 6, the differentiated BMDMs were detached, counted, and reseeded to the cell culture plate for subsequent experiments.

Instrument	BD LSR Fortessa X-20 were used for data collection
Software	Flowjo V10
Cell population abundance	Single-cell suspensions (1 x 10^6 cells) from the tumors, spleen and bone marrow were used.

Gating strategy

Supplementary Figure 4a
Neutrophils were defined as FSC-SSC subset/single cell/single cell/live cell/CD45.2+/Ly6G
TAMs were defined as FSC-SSC subset/single cell/single cell/live cell/CD45.2+/Ly6G-/F4/80+CD11b+
B cells were defined as FSC-SSC subset/single cell/single cell/live cell/CD45.2+/F4/80-CD11b-/CD19+
CD3 T cells were defined as FSC-SSC subset/single cell/single cell/live cell/CD45.2+/F4/80-CD11b-/CD19-CD3+
CD8 T cells were defined as FSC-SSC subset/single cell/single cell/live cell/CD45.2+/F4/80-CD11b-/CD19-CD3+/CD8+
CD4 T cells were defined as FSC-SSC subset/single cell/single cell/live cell/CD45.2+/F4/80-CD11b-/CD19-CD3+/CD4+
Naive CD8 T cells were defined as FSC-SSC subset/single cell/single cell/live cell/CD45.2+/F4/80-CD11b-/CD19-CD3+/CD8+/CD44-CD62L+
Effector/Effector memory CD8 T cells were defined as FSC-SSC subset/single cell/single cell/live cell/CD45.2+/F4/80-CD11b-/CD19-CD3+/CD8+/CD44+CD62L-
Central memory CD8 T cells were defined as FSC-SSC subset/single cell/single cell/live cell/CD45.2+/F4/80-CD11b-/CD19-CD3+/CD8+/CD44+CD62L+

Supplementary Figure 4b
iNOS+ TAMs were defined as FSC-SSC subset/single cell/single cell/live cell/CD45.2+/F4/80+CD11b+/iNOS+
Arg1+ TAMs were defined as FSC-SSC subset/single cell/single cell/live cell/CD45.2+/F4/80+CD11b+/Arg1+

Supplementary Figure 4c
IFN-g+ CD8 T cells were defined as FSC-SSC subset/single cell/single cell/live cell/CD4-CD8+/TNF-a-IFN-g+
TNF-a+ CD8 T cells were defined as FSC-SSC subset/single cell/single cell/live cell/CD4-CD8+/TNF-a+IFN-g-

Supplementary Figure 7c
F4/80+CD11b+ TAMs were defined as FSC-SSC subset/single cell/single cell/live cell/F4/80+CD11b+
TIM3+PD-1+ TAMs were defined as FSC-SSC subset/single cell/single cell/live cell/F4/80+CD11b+/TIM3+PD-1+

Supplementary Figure 11a
F4/80+CD11b+ TAMs were defined as FSC-SSC subset/single cell/single cell/live cell/F4/80+CD11b+/pHrodo Red

Supplementary Figure 11d
H-2Kb SIINFEKL were defined as FSC-SSC subset/single cell/single cell/live cell/F4/80+CD11b+/H2-Kb SIINFEKL

Supplementary Figure 11e
CFSE+ OT-I T cells were defined as FSC-SSC subset/single cell/single cell/live cell/CD8+/CFSE+

Supplementary Figure 14
CD80+ or CD86+ or I-A/I-E+ BMDMs were defined as FSC-SSC subset/single cell/single cell/live cell/F4/80+CD11b+/CD80 or CD86 or I-A/I-E

Supplementary Figure 15
CXCL9+SPP1+ BMDMs were defined as FSC-SSC subset/single cell/single cell/live cell/F4/80+CD11b+/CXCL9+SPP1+

Supplementary Figure 17d
Exhausted CD8+ T cells were defined as FSC-SSC subset/single cell/single cell/live cell/CD45.2+/CD8+

Supplementary Figure 22b, c
iNOS+Arg1+ Macrophages were defined as FSC-SSC subset/single cell/single cell/live cell/CD45.2+/F4/80+CD11b+

Supplementary Figure 22d
CXCR3+CX3CR1+ CD8+ T cells were defined as FSC-SSC subset/single cell/single cell/live cell/CD45.2+/CD8+

☒ Tick this box to confirm that a figure exemplifying the gating strategy is provided in the Supplementary Information.
